# Subthreshold Thermal Stress Aggravates Methamphetamine-Induced Cardiomyocyte Pyroptosis via the Mitochondrial ROS/BAX/mtDNA/NLRP3 Pathway

**DOI:** 10.3390/ijms27115000

**Published:** 2026-05-31

**Authors:** Mengmeng Wang, Congcong Hou, Menglian Hu, Dan Zhou, Xintao Wang, Mingyang Jin, Chunling Ma, Jianhong Shi, Zhiyu Ni

**Affiliations:** 1 Clinical Medical College, Hebei University, Baoding 071000, China; wmm0527@126.com; 2 College of Forensic Medicine, Hebei Medical University, Shijiazhuang 050017, China; 18700967@hebmu.edu.cn (C.H.); hml7665@163.com (M.H.); 18780416834@163.com (D.Z.); 18901784@hebmu.edu.cn (X.W.); jinmingyang1891@126.com (M.J.); chunlingma@hebmu.edu.cn (C.M.); 3 Hebei Key Laboratory of Forensic Medicine, College of Forensic Medicine, Hebei Medical University, Shijiazhuang 050017, China; 4 Collaborative Innovation Center of Forensic Medical Molecular Identification, Hebei Medical University, Shijiazhuang 050017, China; 5 Research Unit of Digestive Tract Microecosystem Pharmacology and Toxicology, Chinese Academy of Medical Sciences, Shijiazhuang 050017, China

**Keywords:** methamphetamine, subthreshold thermal stress, cardiomyocyte pyroptosis, mitochondrial DNA, BCL2-associated X, reactive oxygen species, mitochondria-targeted antioxidant, mitoquinone

## Abstract

Methamphetamine (METH)-induced cardiomyocyte injury is the leading cause of mortality beyond acute intoxication. METH abuse often occurs in crowded, poorly ventilated environments, and even moderately high ambient temperatures exacerbate METH-related cardiovascular emergencies. However, the underlying mechanisms by which environmental factors drive the progression of cardiac diseases remain poorly understood. This study modeled the real-world scenario in vivo by exposing mice to METH under normothermic condition (NC, 22 °C) or subthreshold thermal stress (STS, 28 °C, a mild thermal challenge for mice) conditions, and in vitro by using H9c2 cardiomyocytes exposed to METH at 37 °C or 39 °C. STS significantly potentiated METH-induced cardiac dysfunction, mitochondrial ultrastructural damage, and oxidative stress (*p* < 0.05). Mechanistically, the co-exposure impaired mitochondrial respiratory chain complex I and led to excessive mitochondrial ROS (mtROS) production, activating the pro-apoptotic protein BAX, causing mitochondrial outer membrane (MOM) permeabilization and the cytosolic release of mitochondrial DNA (mtDNA). Cytosolic mtDNA-mediated NLRP3 inflammasome activation subsequently executed cardiomyocyte pyroptosis via caspase-1/Gasdermin D (*p* < 0.05). Crucially, the mitochondria-targeted antioxidant mitoquinone (MitoQ) substantially attenuated the aggravated cardiotoxicity by scavenging the initial mtROS (*p* < 0.05), thereby preventing the activation of the downstream BAX/mtDNA/NLRP3 axis. These findings provide evidence for a defined signaling basis for this drug-environment interaction and highlight mitochondrial redox modulation as a potential therapeutic strategy for psychostimulant-associated cardiovascular injury.

## 1. Introduction

Methamphetamine (METH) abuse poses a growing global health burden, with cardiovascular complications representing a major contributor of morbidity and mortality among METH users. Beyond acute overdose, METH-induced cardiomyocyte injury is characterized by progressive myocardial injury, ventricular dysfunction, and eventual heart failure [1,2,3]. While these clinical manifestations are well documented, the molecular mechanisms that translate METH exposure into sustained cardiomyocyte damage remain incompletely understood, particularly under conditions that mimic real-world environmental stress, significantly hampering the development of targeted therapeutic interventions [4,5].

Environmental stressors, particularly elevated ambient temperature, are a critical but often overlooked factor. Understanding how environmental factors interact with either single or multiple drugs to exacerbate injury in specific organs, such as the heart, is critically important, especially given the higher mortality and more complex toxicities driven by the current polysubstance abuse crisis [6]. METH abuse often occurs in specific environmental settings, such as crowded, poorly ventilated recreational venues [7,8]. Epidemiological data from California showed that even modest increases in ambient temperature were associated with a significant rise in emergency department visits for METH-related cardiovascular emergencies [7], which suggests that mild environmental thermal stress can synergistically exacerbate METH cardiotoxicity. In our previous studies [9], this condition has been referred to as high ambient temperature (HAT, 28 °C) to denote its physiological impact. Here, to more precisely capture its nature as a relevant yet non-extreme environmental challenge commonly encountered in abuse settings, we define it as “subthreshold thermal stress (STS, 28 °C)”. This temperature was specifically chosen because, while perceived as comfortable by humans, it imposes a detectable thermal load on mice whose thermoneutral zone is 30–32 °C [10]. However, whether and how this thermal stress directly amplifies METH-induced cardiotoxicity at the cellular and molecular levels remains largely unexplored. Understanding this drug–environment interaction is essential for defining the pathological context in which METH-induced cardiac injury occurs.

Recent studies have confirmed that pyroptosis contributes to cardiotoxicity under diverse stress conditions [11,12,13]. In cardiomyocytes, activation of the NLRP3 inflammasome and subsequent caspase-1/GSDMD signaling can drive membrane rupture, cell osmotic swelling, and the release of proinflammatory factors such as interleukin-1β [14,15]. While pyroptosis has been linked to METH-related neurotoxicity, the specific upstream signals that trigger NLRP3 inflammasome activation in cardiomyocytes remain elusive, limiting a mechanistic understanding of how this inflammatory death program is initiated.

Mitochondria are central regulators of cellular stress responses and have emerged as key signaling hubs linking cellular stress to innate immune activation [16,17]. In particular, mitochondrial DNA leakage into the cytosol can act as a potent damage-associated molecular pattern (DAMP) to directly activate the NLRP3 inflammasome [18,19]. This process is closely associated with mitochondrial outer membrane (MOM) permeabilization, which can be mediated by the pro-apoptotic protein BAX [20,21]. While BAX/BAK can mediate apoptosis, our study focuses on their role in mtDNA release, which is a known trigger for the NLRP3 inflammasome and subsequent pyroptosis. The resulting pro-inflammatory cell death (pyroptosis) is the key driver of the observed inflammatory cardiac injury, distinguishing it from the generally anti-inflammatory nature of apoptosis. Notably, mitochondrial abnormality [12] and oxidative stress [22,23] have been reported in response to METH exposure, but whether a mitochondria-to-inflammasome signaling axis operates in METH-induced cardiotoxicity—especially under thermal stress—remains unclear, impeding a comprehensive understanding of the mechanisms underlying METH-induced cardiac injury [24].

In this study, we aimed to illustrate the mechanistic link connecting mitochondrial damage to inflammasome-driven pyroptosis in MIC, with a specific focus on the aggravating role of thermal stress. Using MIC models in vivo and in vitro under thermal stress, we identify and functionally validate the mitochondrial ROS-driven BAX-mtDNA signaling cascade that promotes NLRP3-dependent pyroptosis in cardiomyocytes. Furthermore, we demonstrate that Mitochondria-targeted antioxidant (mitoquinone, mitoQ) effectively disrupts this pathogenic axis and alleviates cardiac dysfunction. These findings provide novel and translational insights by mechanistically linking a prevalent environmental stressor (subthreshold heat) to mitochondrial injury, and inflammatory cell death in METH cardiotoxicity, suggesting mitochondrial redox modulation as a potential and promising therapeutic strategy.

## 2. Results

### 2.1. Subthreshold Thermal Stress Exacerbates METH-Induced Cardiac Injury

**Figure 1:**
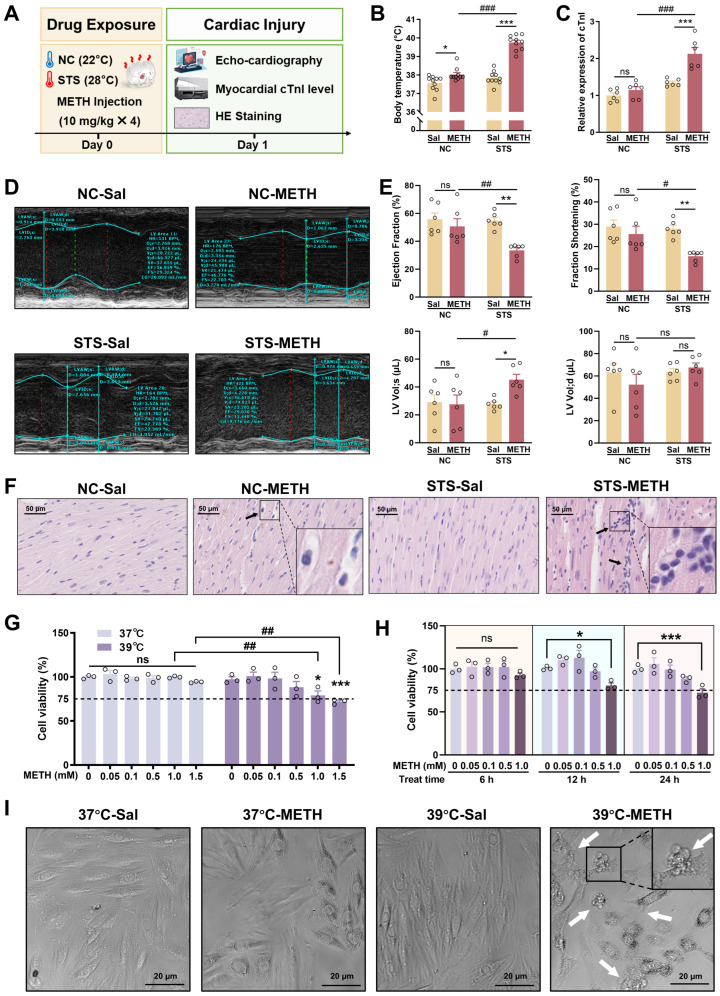
Subthreshold thermal stress exacerbates methamphetamine-induced cardiac injury. (**A**) Experimental timeline of drug administration and cardiac functional assessments. Panels show core temperature measurements ((**B**); *n* = 10), myocardial cTnI level ((**C**); *n* = 6), representative image of echocardiography and cardiac function indicators ((**D**,**E**); *n* = 6, the green and red dotted lines respectively represent the end-diastolic and end-systolic inner diameters of the left ventricle), and representative HE staining ((**F**); scale bar = 50 μm) in the NC-Sal, STS-Sal groups, NC-METH and STS-METH groups. (**G**,**H**) CCK8 assays under varying concentrations of METH (0.05–1.5 mM), normal (37 °C) and high (39 °C) ambient temperatures, and different treatment time (n = 3, the dotted lines represent that the cell survival rate is 75%). Representative images of bubble-like protrusions under light microscopy ((**I**); scale bar = 20 μm) in H9c2 cells exposed to METH. Data presented as mean ± SEM. */# *p* < 0.05; **/## *p* < 0.01; ***/### *p* < 0.001; ns, not significant.

Consistent with our previous experimental timeline (Figure 1A), mice received four intraperitoneal (i.p.) injections of 10 mg/kg METH at 2 h intervals at normothermic condition (NC, 22 °C) and subthreshold thermal stress (STS, 28 °C) ambient temperature. Controls were administered equivalent saline volumes under the same thermal conditions [9]. Two-way ANOVA revealed significantly exacerbated hyperthermia in METH/STS co-exposed mice compared to METH/NC controls (*p* < 0.001, Figure 1B).

The thermosensitive cardiotoxicity of METH was determined by quantifying myocardial injury biomarkers and cardiac functional parameters. METH/STS co-exposure markedly elevated myocardial cTnI levels compared to other groups (Figure 1C). Echocardiography revealed temperature-dependent systolic dysfunction in METH/STS mice, as evidenced by reduced EF, FS and LV Vol;s (*p* < 0.001, Figure 1D,E). However, the diastolic parameters (LV Vol;d) remained unchanged, indicating selective left ventricular systolic impairment under combined thermal and pharmacological stress. Histopathology (HE staining) revealed inflammatory infiltrates in co-exposed cardiac tissues (Figure 1F), indicating METH/STS activates myocardial inflammatory njury.

To investigate the direct role of METH and ambient temperature on cardiomyocyte injury, H9c2 cardiomyocytes were exposed to increasing METH concentrations (0.05–1.5 mM) under normal (37 °C) and high (39 °C) ambient temperatures. First, H9c2 cells directly exposed to METH and normal ambient temperature (37 °C) did not undergo cell death in vitro (Figure 1G, Left). In contrast, CCK8 assays revealed that METH induced a dose-dependent reduction in H9c2 cell viability under high (39 °C) ambient temperatures (Figure 1G, Right). Further CCK8 assays illustrated that H9c2 cells viability was significantly reduced when cells were exposed with 1.0 mM METH at 39 °C for 12 or 24 h instead of 6 h, confirming that the time-dependent cytotoxic effect of METH (Figure 1H). Furthermore, bright-field microscopy revealed that METH (1.0 mM) induced extensive cell rounding, membrane blebbing, detachment and characteristic bubble-like protrusions (a morphological feature commonly associated with pyroptosis, though partially overlapping with other cell death modalities) in H9c2 cells (Figure 1I). These results indicate that high ambient temperature exacerbates methamphetamine-induced cardiomyocyte injury and left ventricular systolic dysfunction.

### 2.2. METH/STS Triggers Cardiomyocyte Pyroptosis In Vivo and In Vitro

Transcriptomic analysis of left ventricular tissues was performed to investigate the mechanism underlying cardiomyocyte toxicity following METH exposure. Principal component analysis (PCA) revealed substantial overlap between the NC-Sal and STS-Sal groups, while both groups were clearly separated from the NC-METH and STS-METH groups (Figure S1A). Furthermore, differential expression analysis (*p* ≤ 0.05 and |log2 Fold Change| ≥ 1.0) identified 989 differentially expressed genes (DEGs, 571 upregulated and 418 downregulated) between the STS-Sal and STS-METH groups (see volcano plot in Figure S1B).

**Figure 2:**
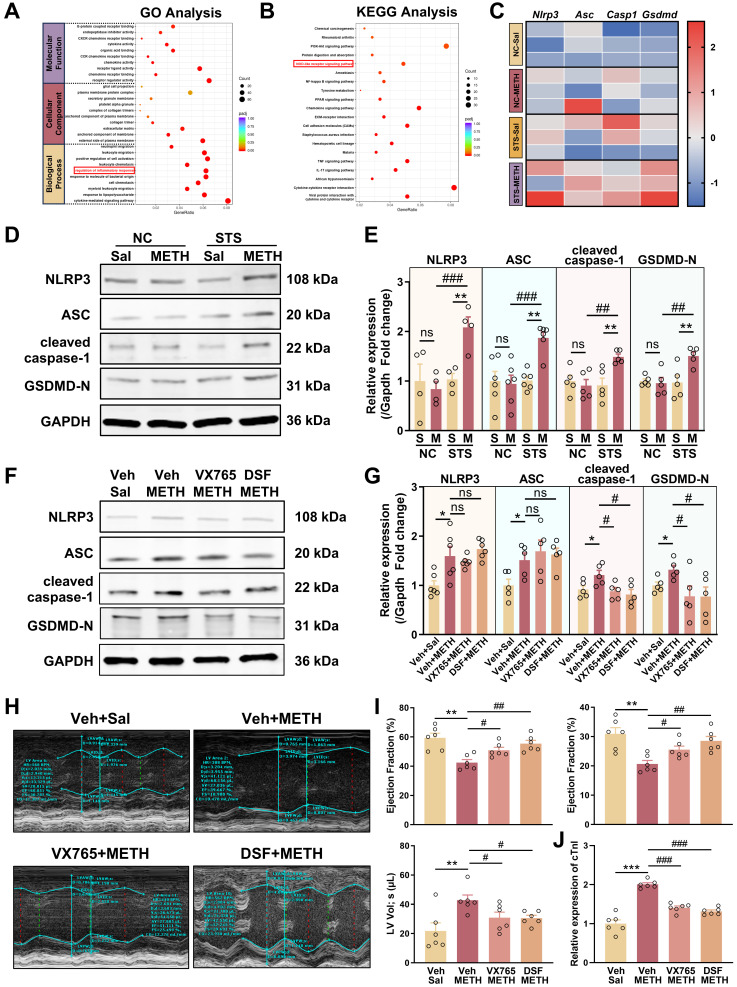
METH/STS induces cardiomyocyte pyroptosis and inflammatory injury in vivo. (**A**,**B**) GO and KEGG analyses between the STS-Sal and STS-METH groups. The heatmap ((**C**); *n* = 3) for visualizing the mRNA levels of the pyroptosis-related genes, Western blot analysis ((**D**,**E**); *n* = 4–6) of pyroptosis effector proteins expression under different conditions. Western blot analysis of pyroptosis effector proteins expression ((**F**,**G**); *n* = 5–6), representative graphs of echocardiography and cardiac function indicators ((**H**,**I**); *n* = 6, the green and red dotted lines respectively represent the end-diastolic and end-systolic inner diameters of the left ventricle), myocardial cTnI levels ((**J**); *n* = 6) after METH/STS exposure with or without pyroptosis inhibitor pretreatment (VX765 or DSF). Data presented as mean ± SEM. */# *p* < 0.05; **/## *p* < 0.01; ***/### *p* < 0.001; ns, not significant.

Gene Ontology (GO) and Kyoto Encyclopedia of Genes and Genomes (KEGG) analyses revealed that the DEGs were enriched in pathways such as the cytokine-mediated signaling pathway, regulation of inflammatory response, and NOD-like receptor signaling pathway (Figure 2A,B). GO mining further indicated METH/STS-specific alterations in IL-1 response and Il-1β production (Table S1). IL-1β regulation involves transcriptional activation, inflammasome-dependent or independent caspase cleavage, and multiple secretory pathways [25]; therefore, DEGs encoding inflammasome and IL-1β regulators were analyzed. The heatmap revealed altered mRNA levels of pyroptosis-related genes in STS-METH myocardium tissues compared to other groups (Figure 2C). Immunoblotting and ELISA confirmed upregulated pyroptosis effector proteins (Figure 2D,E) and IL-1β (Figure S1C) in the STS-METH group.

Pharmacological inhibition with caspase-1 inhibitor (VX765) or GSDMD inhibitor (disulfiram, DSF) significantly downregulated cleaved caspase-1 and GSDMD-N expression compared to the STS-METH group (Figure 2F,G). It should be noted that potential off-target effects of VX765 and DSF cannot be fully excluded, which should be considered when interpreting the findings. However, their protective effects in our model are consistent with the proposed pyroptosis pathway. Echocardiography indicated that VX765 or DSF pretreatment improved EF, FS, and LV Vol;s in mice from the STS-METH group (Figure 2H,I), while decreasing the levels of cTnI (Figure 2J) and IL-1β (Figure S1D), as well as attenuated inflammatory infiltration in myocardial tissue (Figure S1E).

Meanwhile, immunoblotting confirmed that the co-treatment of METH (1.0 mM) and STS upregulated pyroptosis effectors (Figure S1F,G). Pharmacological inhibition with VX765 or DSF significantly downregulated the levels of cleaved caspase-1 and GSDMD-N compared to the Veh+METH group (Figure S1H,I) and cell death (Figure S1J), suggesting that METH/STS induces cardiomyocyte morphological alterations and pyroptosis in vitro.

Collectively, these results demonstrate that high ambient temperature dramatically exacerbates METH-induced cardiotoxicity in vivo and in vitro, which is mechanistically linked to the NLRP3 inflammasome activation and subsequent induction of pyroptosis.

### 2.3. METH/STS Triggers Cardiomyocyte Pyroptosis via mtDNA Escape

**Figure 3:**
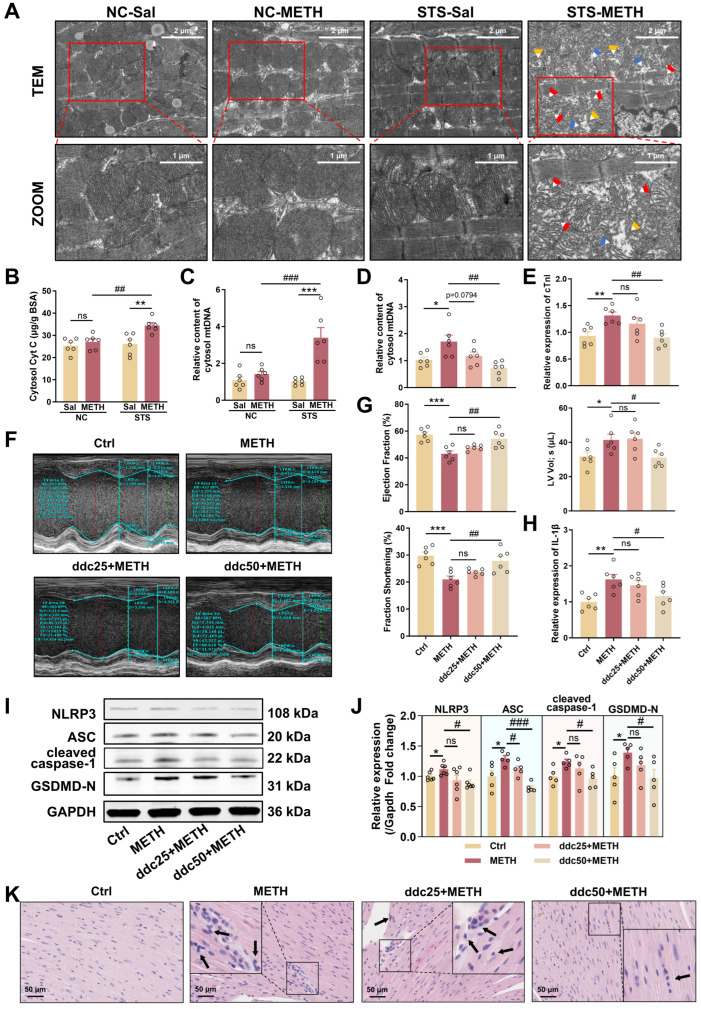
METH/STS triggers cardiomyocyte pyroptosis via mtDNA escape. Representative TEM images ((**A**), scale bar = 1 or 2 μm), quantitative analysis of cytosol Cyt C (**B**) and mtDNA content (**C**) exposed to METH and/or high ambient temperature (n = 6). Quantitative analysis of mtDNA content ((**D**); *n* = 6), myocardial cTnI level ((**E**); *n* = 6), representative graphs of echocardiography and cardiac function indicators ((**F**,**G**); *n* = 6, the green and red dotted lines respectively represent the end-diastolic and end-systolic inner diameters of the left ventricle), myocardial IL-1β level ((**H**); *n* = 6), pyroptosis effector proteins expression ((**I**,**J**); *n* = 5), and representative HE staining ((**K**); scale bar = 50 μm) under METH/STS co-exposure after ddc pretreatment (25 or 50 mg/kg). Data presented as mean ± SEM. */# *p* < 0.05; **/## *p* < 0.01; ***/### *p* < 0.001; ns, not significant.

Mitochondria, as central regulatory organelles maintaining cardiomyocyte homeostasis, exhibit functional abnormalities that can lead to severe cardiomyocyte dysfunction [26]. Transmission electron microscopy (TEM) revealed mitochondrial ultrastructural damage in myocardial tissues from the STS-METH group, including cristae dissolution (red arrows), vacuolation (yellow arrows) and membrane rupture (blue arrows) (Figure 3A). Further GO analysis indicated that METH/STS induced Cyt C release from mitochondria and regulation of mitochondrial membrane potential (Table S2).

Furthermore, METH/STS significantly promoted cytosolic translocation of Cyt C (Figure 3B) and mitochondrial DNA (mtDNA, Figure 3C), indicating that METH/STS co-exposure induced loss of membrane integrity, and mtDNA release. Cytosolic mtDNA is involved in activating inflammasome-associated pyroptosis, so the mtDNA polymerase inhibitor dideoxycytidine (ddc) was employed to explore the effects of METH exposure on mtDNA distribution. Ddc (50 mg/kg) inhibited cytosolic mtDNA release (Figure 3D), while reduced cTnI (Figure 3E), ameliorated systolic dysfunction (Figure 3F,G), IL-1β (Figure 3H), pyroptosis effectors (Figure 3I,J), and inflammatory infiltration (Figure 3K).

We next aimed to determine whether the mtDNA escape is involved in METH/STS-induced cardiotoxicity in vitro. In H9c2 cells, METH/STS induced mPTP opening (Figure S2A)and mtDNA translocation (Figure S2B,C). The CCK-8 assay results confirmed that ddc (10–160 μM, 24 h) did not affect cell viability (Figure S2D). Furthermore, ddc (160 μM) reduced cellular mtDNA by about 50% (Figure S2E) and attenuated METH/STS-induced cell death (Figure S2F), cytosolic mtDNA accumulation (Figure S2G), pyroptosis protein expression (Figure S2H,I). Therefore, METH/STS-induced mtDNA escape triggers myocardial pyroptosis and injury in vivo and in vitro.

### 2.4. METH/STS Activates Cardiomyocyte Pyroptosis via BAX-Mediated mtDNA Escape

**Figure 4:**
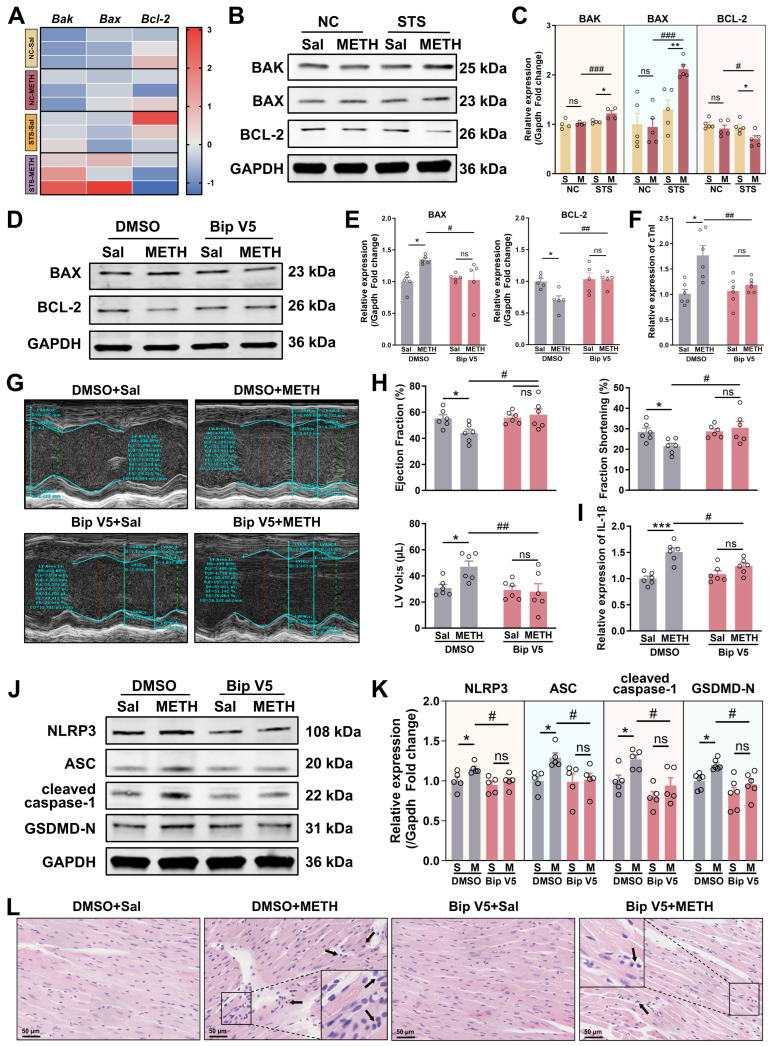
METH/STS activates cardiomyocyte pyroptosis via BAX-mediated mtDNA escape. Heatmaps for visualizing the mRNA levels of the pyroptosis-related genes exposed to METH and/or high ambient temperature ((**A**); *n* = 3). Immunoblotting analysis of BCL-2-related protein expression exposed to METH and/or high ambient temperature ((**B**,**C**); *n* = 5). Immunoblotting analysis of BCL-2-related protein expression ((**D**,**E**); *n* = 5), myocardial cTnI level ((**F**); *n* = 6), representative graphs of echocardiography and cardiac function indicators ((**G**,**H**); *n* = 6, the green and red dotted lines respectively represent the end-diastolic and end-systolic inner diameters of the left ventricle), myocardial IL-1β level ((**I**); *n* = 6), pyroptosis effector protein expression ((**J**,**K**); *n* = 5–6), and representative HE staining ((**L**); scale bar = 50 μm) after METH/STS exposure with or without Bip V5 pretreatment. Data presented as mean ± SEM. */# *p* < 0.05; **/## *p* < 0.01; ***/### *p* < 0.001; ns, not significant.

GO analysis indicated that METH/STS co-exposure is associated with mitochondrial apoptotic changes and alterations in mitochondrial outer membrane permeability (MOMP), both of which are hallmarks of programmed cell death (Table S2). The activation of the key molecular switch, BAK/BAX, regulates the mechanism underlying mtDNA escape by remodelling the mitochondrial outer membrane (MOM) to generate nanoscale pores known as mitochondrial apoptosis-inducing channels [27,28]. Transcriptomics analysis revealed that METH induced Bak/Bax upregulation and Bcl-2 downregulation (Figure 4A). Moreover, protein levels were confirmed by immunoblotting, indicating that METH/STS co-exposure induced dysregulation of BCL-2-related protein family expression (Figure 4B,C). In contrast, BAX inhibitor peptide V5 (Bip V5) pretreatment normalized BAX and BCL-2 expression (Figure 4D,E), reduced cTnI levels (Figure 4F), improved left ventricular systolic function (Figure 4G,H), cytosolic Cyt C/mtDNA content (Figure S3A,B), IL-1β (Figure 4I), pyroptosis proteins (Figure 4J,K), and inflammatory infiltration (Figure 4L).

In vitro, METH/STS co-exposure also upregulated BAK/BAX and downregulated BCL-2 in H9c2 cells (Figure S3C,D). However, Bip V5 pretreatment significantly mitigated METH/STS-induced BAX/BCL-2 dysregulation (Figure S3C,D), cell death (Figure S3E), mPTP opening (Figure S3F), and cytosolic mtDNA translocation (Figure S3G) in H9c2 cells. In addition, Bip V5 pretreatment also downregulated the expression of pyroptosis effectors (Figure S3H,I). These results indicate that METH/STS activates cardiomyocyte pyroptosis via BAX-mediated mtDNA escape.

### 2.5. METH/STS Inhibits Mitochondrial Respiratory Chain Complex I Activity and Induces Oxidative Stress

**Figure 5:**
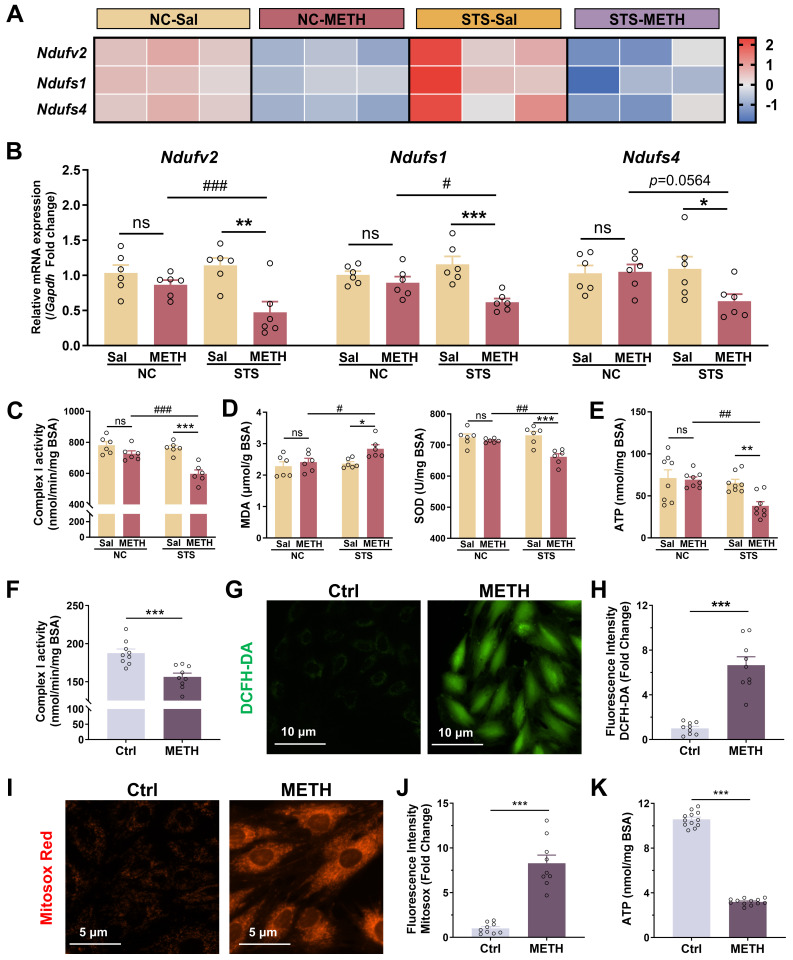
METH/STS inhibits mitochondrial respiratory chain complex I activity and induces oxidative stress. The heatmap for visualizing ((**A**); *n* = 3) and the relative mRNA expression levels ((**B**); *n* = 6) of the mitochondrial complex I-related genes (*Ndufv2*/*Ndufs1*/*Ndufs4*), and quantitative analysis of complex I activity, MDA, SOD and ATP levels exposed to METH and/or STS ((**C**–**E**); *n* = 6 or 8). Quantitative analysis of complex I activity ((**F**); *n* = 9), representative graphs and fluorescence intensity of cellular ROS ((**G**,**H**); *n* = 9) and mtROS ((**I**,**J**); *n* = 9), and ATP levels ((**K**); *n* = 12) in METH/STS-challenge H9c2 cells. Data presented as mean ± SEM. */# *p* < 0.05; **/## *p* < 0.01; ***/### *p* < 0.001; ns, not significant.

Mitochondrial respiratory chain complex I serves as the entry point for electrons from NADH into the electron transport chain (ETC) and represents a major source of mitochondrial ROS (mtROS), which have been shown to be critical in the abnormal activation of the BCL-2-related protein family [29,30]. Transcriptome and qPCR analysis of the left ventricular tissue revealed altered mRNA levels of mitochondrial respiratory chain complex I-related genes (*Ndufv2*/*Ndufs1*/*Ndufs4*) in STS-METH myocardium tissues compared to other groups (Figure 5A,B). METH/STS co-treatment resulted in suppressive complex I activity (Figure 5C), increased MDA (Figure 5D, Left) and decreased SOD (Figure 5D, Right), and ATP (Figure 5E) levels, indirectly indicating METH/STS-induced oxidative stress and energy disruption. We next aimed to explore whether METH/STS co-exposure could trigger similar mitochondrial dysfunction described above in vitro. In H9c2 cells, METH/STS also induced complex I inactivation (Figure 5F), ROS overproduction in the whole cells (Figure 5G,H) and mitochondria (Figure 5I,J) and ATP depletion (Figure 5K).

### 2.6. MitoQ Ameliorates METH/STS-Induced Cardiomyocyte Pyroptosis by Inhibiting ROS/BAX/mtDNA Escape

**Figure 6:**
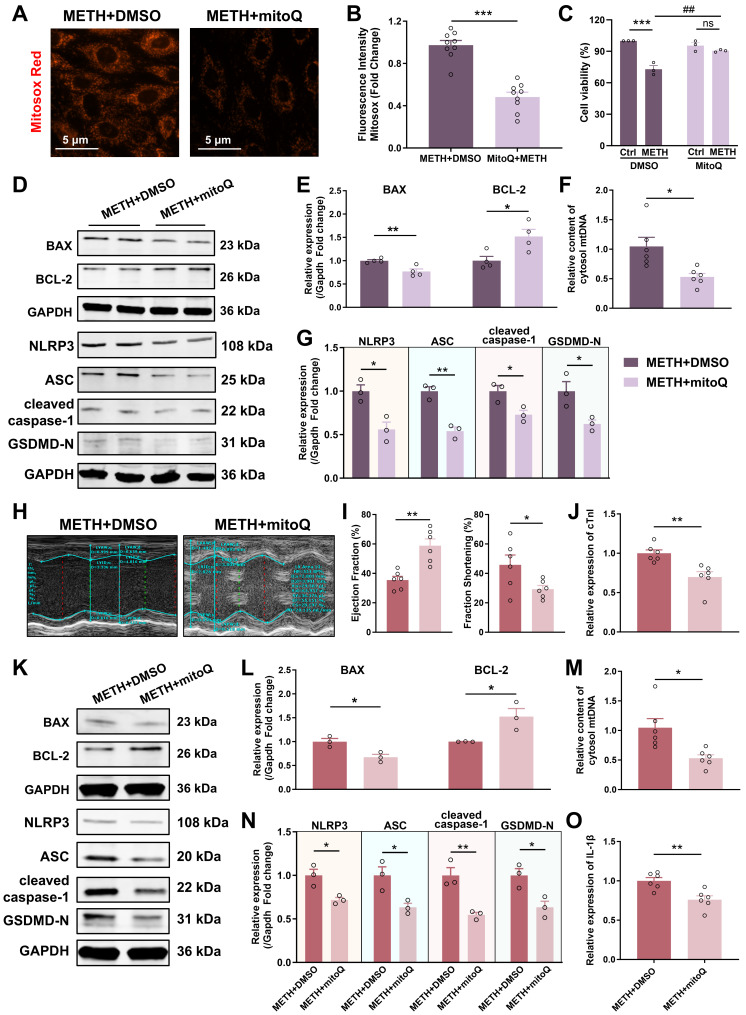
MitoQ ameliorates METH/STS-induced cardiomyocyte pyroptosis by inhibiting ROS/BAX/mtDNA escape. Representative graphs and fluorescence intensity of mtROS ((**A**,**B**); *n* = 9), cell viability ((**C**); *n* = 3), BAX/BCL-2 expression ((**D**,**E**); *n* = 4), cytosol mtDNA content ((**F**); *n* = 6) and pyroptosis effector proteins expression ((**D**,**G**); *n* = 3) in METH/STS-challenge H9c2 cells with/without MitoQ intervention. Representative graphs of echocardiography and cardiac function indicators ((**H**,**I**); *n* = 6, the green and red dotted lines respectively represent the end-diastolic and end-systolic inner diameters of the left ventricle), myocardial cTnI level ((**J**); *n* = 6), BAX/BCL-2 expression ((**K**,**L**); *n* = 3), cytosol mtDNA content ((**M**); *n* = 6), pyroptosis effector proteins expression ((**K**,**N**); *n* = 3), and IL-1β ((**O**); *n* = 6) after METH/STS exposure with or without mitoQ intervention. Data presented as mean ± SEM. * *p* < 0.05; **/## *p* < 0.01; *** *p* < 0.001; ns, not significant.

**Figure 7:**
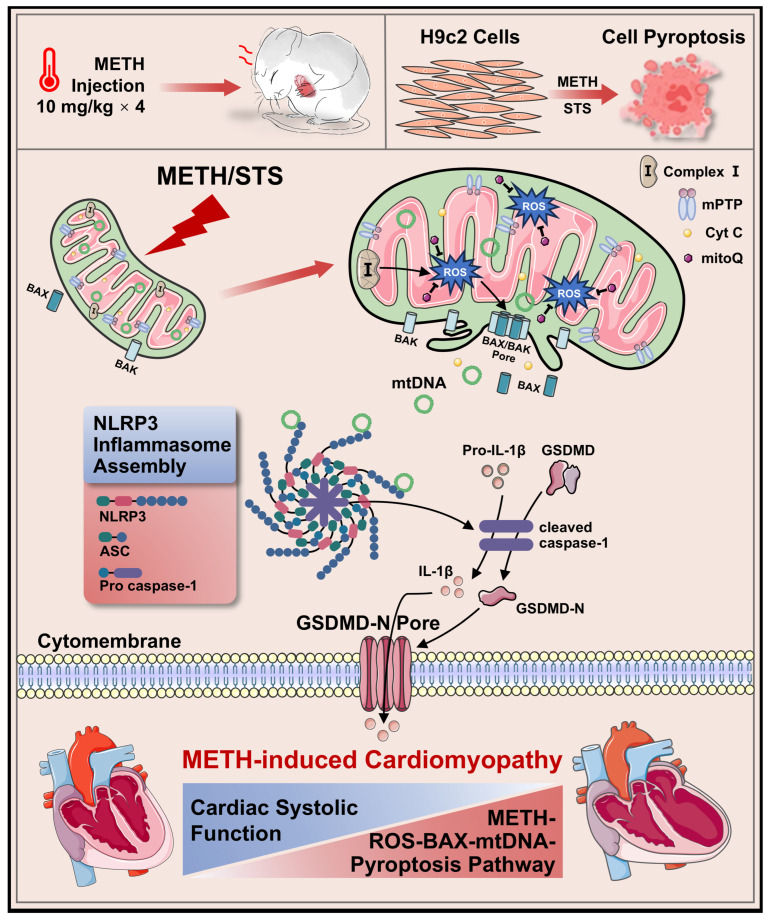
Subthreshold Thermal Stress Aggravates Methamphetamine-Induced Cardiomyocyte Pyroptosis via the Mitochondrial ROS/BAX/mtDNA/NLRP3 Pathway. The graphical abstract provides an overview of the core molecular pathways involved in METH/STS causing cardiac damage. METH/STS exposure induces a burst of mitochondrial ROS by inhibiting mitochondrial respiratory chain complex I, activates BAX and causes mtDNA leakage into the cytoplasm, and therefore activates the NLRP3 inflammasome and caspase-1/GSDMD-dependent pyroptosis, while MitoQ reversed METH/STS-induced myocardial injury by alleviating oxidative stress. The graphical abstract was created using BioRender.com.

MitoQ, composed of triphenylphosphine (TPP) cation and coenzyme Q10, could specifically enrich in the mitochondrial matrix and directly neutralize mitochondrial ROS, and therefore regulating oxidative stress and corresponding cell functions [31]. Next, mitoQ was used to examine the effect of METH/STS-induced ROS abnormalities on myocardial injury. As expected, mitoQ intervention (250 nM, 12h after METH/STS treatment) reversed METH/STS-induced mtROS overproduction (Figure 6A,B) and cell death (Figure 6C), while attenuating the aberrant BAX/BCL-2 expression (Figure 6D,E), cytosolic mtDNA accumulation (Figure 6F), and pyroptosis effectors expression (Figure 6D,G) in H9c2 cells. In vivo, mitoQ intervention (10 mg/kg, twice daily after METH/STS treatment) improved left ventricular systolic function (Figure 6H,I), reduced cTnI levels (Figure 6J), normalized BAX/BCL-2 expression (Figure 6K,L), cytosolic Cyt C/mtDNA content (Figure 6M), pyroptosis proteins (Figure 6K,N) and IL-1β (Figure 6O). These results suggest that, as a mitochondria-targeted antioxidant, MitoQ reversed METH/STS-induced cardiomyocytes pyroptosis by attenuating oxidative stress and BAX-mediated mtDNA escape (Figure 7).

## 3. Discussion

Here, we demonstrate that subthreshold thermal stress (STS), a condition prevalent in real-world methamphetamine (METH) abuse scenarios, acts as a critical synergistic factor that aggravates cardiotoxicity through the ROS/BAX/mtDNA axis. Furthermore, the mitochondria-targeted antioxidant mitoQ confers substantial cardioprotection by quenching mitochondrial ROS (mtROS), thereby intercepting this cascade and attenuating cardiac inflammation and dysfunction.

METH, a phenethylamine-derived psychostimulant with high addictive potential, has been shown to induce progressive myocardial injury, cardiac dysfunction, and increased risk of sudden cardiac death [32,33]. Preclinical models employing chronic paradigms reveal that METH promotes pathological cardiac remodeling, characterized by myofibrillar disarray and impaired systolic function [34]. Although the cardiovascular sequelae of METH are extensively studied, the environmental context of recreational psychostimulant use, such as sustained thermal stress in crowded recreational venues (e.g., dance halls, raves), remains frequently overlooked. These environments promote heat-related morbidity through the combined effects of hyperthermia, restricted hydration, and prolonged physical exertion [7,8]. The present study shows that METH exposure induces cardiac pathology in a temperature-dependent manner. To recapitulate the environmental conditions experienced by METH abusers in crowded recreational settings, we selected 28 °C as the ambient temperature for inducing mild thermal stress in mice according to established protocols [9]. This METH regimen (4 × 10 mg/kg, 2 h intervals) models the binge pattern of recreational use. By body surface area conversion, 10 mg/kg in mice approximates 57 mg/70 kg human, consistent with a heavy single binge dose (50–200 mg) [35,36]. The regimen alone under normothermic conditions (22 °C) produces only mild effects, but synergistically exacerbates cardiotoxicity under subthreshold thermal stress (28 °C), precisely capturing the drug-environment interaction. Although 28 °C is perceived as thermoneutral in humans, it imposes a substantial thermal challenge in mice, whose thermoneutral zone ranges from 30 °C to 32 °C—approximately 5 °C higher than that of humans. Subthreshold thermal stress (STS, 28 °C) potentiated METH-induced hyperthermia and precipitated severe cardiac dysfunction with histopathological damage, while equivalent dosing under normothermic ambient temperature (NC, 22 °C) did not. These findings corroborate epidemiological data from California (2005–2019) linking elevated ambient temperatures to increased emergency department visits for METH-related complications, highlighting thermosensitivity in stimulant-using populations [8]. These data underscore the urgent need for harm-reduction strategies in recreational settings where hyperthermia may amplify psychostimulant-induced cardiovascular risk [37]. Our experimental model provides a robust platform for investigating molecular targets underlying METH-associated cardiomyopathy. Further studies should incorporate the detailed profiles of METH users to assess the impact of diverse usage patterns and environmental conditions on drug misuse behavior and cardiac outcomes.

We acknowledge that the concentrations required to induce robust cytotoxicity in vitro (particularly 1.0–1.5 mM) are higher than typical plasma levels reported in acute intoxication cases (which are often in the micromolar range). We provide three key explanations: (1) tissue accumulation: drug concentrations in specific organs (e.g., heart) of chronic abusers may be significantly higher than in plasma due to accumulation; (2) limitation of in vitro models: cell culture systems lack the full pharmacokinetics, metabolism, and tissue distribution of the whole organism, often requiring higher concentrations to model the cumulative pathological effects of chronic exposure seen in vivo; (3) mechanistic exploration: the wider concentration range was used to definitively establish the dose-dependent effect and the underlying molecular pathway in vitro, providing proof-of-principle for the phenomena observed in vivo. We emphasize that the core value of this study is the identification of a novel molecular pathway (mtROS/BAX/mtDNA/NLRP3), whose activation threshold in the context of progressive mitochondrial dysfunction from chronic abuse may be lower.

METH-induced cardiomyocyte injury is partially attributed to Sigma-1 receptors or trace amine-associated receptor 1 activation [38,39]. However, the molecular pathways underlying METH- and hyperthermia-induced cardiac pathology remain poorly defined. Systematic ultrastructural analysis via TEM revealed characteristic cardiomyocyte damage, including membrane rupture and organellar swelling, in METH/STS-exposed hearts, accompanied by upregulated pyroptosis effectors and downstream cytokine IL-1β release. Notably, pretreatment with VX-765 or disulfiram markedly attenuated cardiac pathology, confirming pyroptosis as an STS-dependent mechanism of METH cardiotoxicity. Consistently, direct exposure of H9c2 cardiomyocytes to METH/STS recapitulated cardiotoxic phenotype observed in vivo. To distinguish pyroptosis from other regulated cell death pathways, we relied on both morphological and molecular evidence. The typical membrane ballooning and rupture, coupled with the specific activation of caspase-1 and the pore-forming N-terminal fragment of GSDMD, is definitive for pyroptosis. Moreover, the complete abrogation of cell death and cardiac dysfunction by the caspase-1 inhibitor VX-765 and the GSDMD inhibitor DSF further rules out apoptosis (which is caspase-3/7 dependent) and necroptosis (which is caspase-independent), confirming pyroptosis as the primary pathway. Although our data strongly support pyroptosis as a major outcome, since we did not assess the classic markers of cell apoptosis (cleaved caspase-3 or TUNEL staining), we cannot completely rule out the possible effects of other concurrent or mixed cell death pathways. The translational potential of this pathway is highlighted by the efficacy of VX-765 in phase II clinical trials for inflammatory diseases [40], and disulfiram (DSF), an FDA-approved drug that shows promise in mitigating psychostimulant-induced cardiac injury [41].

Mitochondrial integrity critically regulates inflammatory cell death pathways in cardiomyopathy [42]. We observed that METH/STS exposure induced a spectrum of myocardial pathologies, including mitochondrial ultrastructural damage, oxidative stress, and crucially, cytosolic accumulation of mtDNA. Using the mtDNA polymerase inhibitor ddc, we demonstrated that blocking mtDNA synthesis attenuated pyroptosis and cardiotoxicity caused by METH/STS, identifying cytosolic mtDNA as an essential DAMP in this cascade. Since ddc globally inhibits mtDNA replication and may affect overall mitochondrial function, the observed protective effect highlights the importance of mtDNA in this process. At the same time, it is necessary to acknowledge the secondary effects of ddc on mitochondrial homeostasis, and this non-specific effect may be a potential limitation of this intervention. Although cytosolic oxidized mtDNA preferentially activates the NLRP3 inflammasome during pyroptosis [43], and we also observed a significant activation of oxidative stress, the current data lack an assessment of the level of mtDNA oxidation. Therefore, while our data support a model in which mtDNA acts as a pivotal DAMP, the potential for direct mtDNA-NLRP3 interactions in cardiomyocytes requires further investigation. In addition, the mechanism of mtDNA translocation to the cytosol remains incompletely understood, with current evidence indicating that BAK/BAX oligomerization facilitates MOM macropore formation, enabling mtDNA release via inner membrane (MIM) protrusion [20,27,44]. Our data provide multiple lines of evidence supporting this: inhibition of BAX expression prevented the cytosolic mtDNA escape, and reversed the pyroptosis effectors expression and cardiac inflammatory injury. This establishes that mtDNA escape is not a passive consequence of general mitochondrial disruption, but an active process orchestrated by BAX, thereby creating the specific DAMP (mtDNA) required to license the NLRP3 inflammasome for pyroptosis.

Mitochondrial complex I deficiency has been shown to trigger Bax-dependent neuronal apoptosis through mitochondrial oxidative damage [30]. Our study demonstrates that METH/STS exposure resulted in down-regulation of mitochondrial complex I nuclear coding subunit genes (*Ndufv2*/*Ndufs1*/*Ndufs4*) in cardiomyocytes, suggesting the direct or indirect interference of METH with the mitochondrial biosynthetic program in the nucleus. Our study demonstrates that METH/STS exposure severely impaired mitochondrial complex I activity, a major site of mtROS generation, leading to a profound oxidative stress. We propose that mtROS overproduction acts as a critical redox signal that directly or indirectly promotes mitochondrial conformational changes and oligomerization of BAX, which are key events licensing mtDNA escape. The critical role of ROS as the trigger connecting complex I dysfunction with BAX activation is definitively established by the mitochondria-targeted antioxidantmitoQ. Our data position increased mtROS as a pivotal signaling node rather than a mere epiphenomenon of mitochondrial damage. The effectiveness of MitoQ, even when administered after METH/STS exposure, in blocking the entire BAX/mtDNA/NLRP3 cascade suggests that mtROS actively perpetuates the pro-pyroptotic signal. We propose that initial METH/STS-induced Complex I impairment triggers a “first wave” of mtROS, which then activates BAX, leading to MOMP and mtDNA release. This, in turn, likely exacerbates mitochondrial dysfunction and generates a second wave of ROS, creating a feed-forward loop. Thus, while originating from Complex I dysfunction, mtROS acts as a primary initiator of the downstream inflammatory death pathway. By inhibiting mtROS burst, MitoQ prevented subsequent BAX activation, mtDNA leakage, and NLRP3 inflammasome-driven pyroptosis, thereby conferring cardioprotection. We acknowledge that MitoQ, as a general mitochondrial antioxidant, may exert its cardioprotective effects through multiple redox-sensitive pathways beyond the ROS/BAX/mtDNA axis. Although MitoQ has not been approved by the FDA as a drug yet, several clinical trials have evaluated its efficacy against oxidative damage in various diseases, including cardiovascular disease ([NCT05561556] and [NCT05872139], which highlights the strategic promise of early intervention at the level of mitochondrial oxidative stress.

While this study connects the suppression of nuclear-encoded complex I genes to downstream pathology, the upstream signaling events that initiate this transcriptional repression remain a critical knowledge gap and a key to understanding the earliest phases of METH cardiotoxicity. We propose that METH may suppress mitochondrial biogenesis via one or more of the following mechanisms: First, METH-induced energy crisis may activate sensors like AMPK, which in turn suppress the, master regulator of mitochondrial biosynthesis, PGC-1α, leading to downregulation of its target genes, including *Ndufv2*/*Ndufs1*/*Ndufs4* [45,46]; Second, METH may trigger epigenetic silencing of these via stress-induced recruitment of histone deacetylases (HDACs), histone or DNA methyltransferases to their promoters, establishing a repressive chromatin state [47]. In summary, METH may inhibit the function of the master regulator PGC-1α by activating upstream signaling pathways such as energy stress and epigenetic modification, leading to transcriptional down-regulation of mitochondrial complex I nuclear coding genes.

Several limitations of this study should be acknowledged. First, experiments were conducted exclusively in male mice and H9c2 cardiomyoblasts, leaving sex-dependent effects unexplored and recognizing that H9c2 cells may not fully recapitulate adult primary cardiomyocyte properties. Second, mechanistic conclusions rely heavily on pharmacological inhibitors (e.g., VX765, DSF, ddc, MitoQ), which carry potential off-target effects; for instance, ddc globally impairs mtDNA replication, and MitoQ, as a broad-spectrum mitochondrial antioxidant, may act on multiple redox-sensitive pathways. Third, although BAX/BAK activation was observed, we did not evaluate classical apoptotic markers such as cleaved caspase-3 or TUNEL staining; thus, the contribution of apoptosis cannot be entirely excluded. Nevertheless, our conclusion that pyroptosis is the predominant cell death modality is supported by the significant rescue effects of pyroptosis-specific inhibitors (VX765, DSF).

Notably, similar mitochondrial dysfunction and pyroptosis pathways have been reported in cardiotoxicity induced by other psychostimulants such as 3,4-methylenedioxymethamphetamine (MDMA) [48] and cocaine [49]. MDMA has been shown to induce mitochondrial complex I inhibition and ROS overproduction in cardiomyocytes [50], while cocaine exposure can trigger BAX-mediated mitochondrial permeabilization and NLRP3 inflammasome activation [51]. These findings suggest that the ROS/BAX/mtDNA/NLRP3 axis may represent a common mechanism underlying psychostimulant-related cardiac injury, particularly under thermal stress conditions commonly encountered in recreational settings.

Future studies should delineate the precise upstream signals that suppress complex I core subunit expression and determine whether ROS/BAX/mtDNA axis represents a common mechanism of pyroptosis engaged by other pro-inflammatory and cardiotoxic xenobiotics. In addition, future investigations employing NLRP3 or Caspase-1 genetic knockout models will provide definitive genetic evidence to unequivocally determine whether the mtDNA-mediated cardiotoxicity observed here is dependent on canonical inflammasome signaling, and we are actively pursuing these experiments to complement our current findings.

## 4. Materials and Methods

### 4.1. Animals

All animal experiments utilized male C57BL/6J mice (8-week-old, 20–22 g) purchased from Beijing Vital River Laboratory. The mice were housed under specific pathogen-free conditions with controlled temperature (22 ± 1 °C), humidity (60%), and a 12 h light/dark cycle, with free access to standard chow and water. One hour after the first injection of methamphetamine, the core body temperature of the mice was measured using a lubricated rectal probe. Mice were randomly assigned to groups using a Random number scheme, and that investigators were blinded during all experimental analyses. All experimental procedures were approved by the Institutional Animal Ethics Committee and conducted in accordance with NIH guidelines.

### 4.2. Methamphetamine Treatment

D-methamphetamine (METH) was obtained from the Beijing Municipal Public Security Bureau (Beijing, China). Mice received four injections of 10 mg/kg METH (1 mg/mL, i.p.) at 2 h intervals under normothermic conditions (NC, 22 °C) or subthreshold thermal stress (STS, 28 °C) [52]. STS (28 °C) was chosen to mimic mild environmental hyperthermia encountered during recreational psychostimulant use in crowded venues [7]. Control groups were treated with equal saline following the same administration schedule. Animals were monitored once every hour for signs of distress, including but not limited to: hypothermia (<34.0 °C), loss of righting reflex, persistent stereotypy (e.g., repetitive circling, head weaving), hunched posture with piloerection, and respiratory distress. Any animal meeting one or more of these criteria was immediately removed from the study and euthanized by cervical dislocation under deep isoflurane anesthesia.

### 4.3. Pharmacological Intervention

Belnacasan (VX765, HY-13205; MedChemExpress, Monmouth Junction, NJ, USA) and disulfiram (DSF, HY-B0240; MedChemExpress) were dissolved in 10% DMSO and 90% corn oil and injected (i.p.) at 50 mg/kg per day for three consecutive days prior to METH exposure (VX765+METH and DSF+METH groups) [53,54]. Nucleoside analog 2′3′-dideoxycytidine (ddc, 25 or 50 mg/kg, S1719; Selleck, Houston, TX, USA) and BAX-inhibiting peptide V5 (Bip V5, 2.5 mg/kg, T10463; Target Mol, Boston, MA, USA) were injected (i.p.) once daily for 3 days before METH treatment (ddc25+METH, ddc50+METH and Bip V5+METH groups) [55,56]. MitoQ (MCE, HY-100116A, 10 mg/kg) or vehicle control (1% DMSO) was injected (i.p.) twice daily beginning 12 h after the first METH injection to represent a therapeutic intervention [57].

### 4.4. Echocardiography

Cardiac function was evaluated using the VEVO 770 system (VisualSonics, Toronto, ON, Canada) under isoflurane anesthesia (1.5% in O_2_, R510-22-10, RWD, Shenzhen, China). M-mode recordings were used to measure the parameters of cardiac function.

### 4.5. Hematoxylin-Eosin Staining

Cardiac specimens were embedded in paraffin and cut into 5 μm. After staining with hematoxylin-eosin (H&E) and ethanol dehydration, the sections were observed by using a Leica Aperio CS2 microscope (Leica, Wetzlar, Germany).

### 4.6. Transmission Electron Microscope

Myocardial specimens (1 mm^3^) were fixed in glutaraldehyde and osmium tetroxide, and ethanol dehydration and sliced into 70 nm ultrathin sections. Sections were stained with uranyl acetate and lead citrate, then imaged using a HITACHI TEM system.

### 4.7. RNA-Sequencing

Total RNA was extracted from left ventricular tissues using TRIzol reagent (Takara, San Jose, CA, USA). RNA samples were then used to construct sequencing libraries, which underwent quality control before being subjected to bulk RNA sequencing on the Illumina platform. Then, gene expression counts were generated for myocardial tissues under each drug and ambient condition. Differential analysis was carried out using the “DESeq2” package in R (version 4.3.2). Visualization of the results was achieved through volcano plots and heatmaps.

### 4.8. Cell Culture, Reagents, and Viability

H9c2 cells were maintained in DMEM (C3103; VivaCell, Shanghai, China) supplemented with FBS (C04001; VivaCell) and penicillin/streptomycin (C3420; VivaCell). Cells were exposed to METH (0.05–1.5 mM, 6–24 h) at 37 °C or 39 °C, ddc (10–160 μM, 24 h), Bip V5 (100 μM, 1 h), and mitoQ (250 nM, 12h after METH/STS) [58,59,60]. Cell viability was detected using the CCK-8 kit (C0037; Beyotime, Shanghai, China). For in vitro studies, 39 °C was selected to mimic mild thermal stress in cultured H9c2 cells. This temperature represents a physiologically relevant hyperthermic challenge for mammalian cells in culture, inducing a stress response without causing immediate, non-specific cytotoxicity, and thus serves as a functional equivalent to the 28 °C STS in mice.

### 4.9. Western Blotting

Tissue or cellular lysates were prepared using RIPA buffer (R0010; Solarbio, Beijing, China) with protease inhibitors. Proteins were denatured by boiling for 5 min after adding sample buffer, separated via SDS-PAGE gels, and transferred to PVDF membranes, which were blocked and incubated with primary antibodies, including NLRP3 [ET1610-93; HUABIO, Hangzhou, China], ASC/TMS1 [30641-1-AP; Proteintech, Rosemont, IL, USA], Caspase-1 p20 [ER1905-47; HUABIO], Gasdermin D (N terminal) [ER1901-37; HUABIO], BAX [ET1603-34; HUABIO], BAK [ER1904-10; HUABIO], BCL-2 [ET1702-53; HUABIO], and GAPDH [ET1601-4; HUABIO]. Thereafter, the samples were incubated with LI-COR secondary antibody (C50317). Finally, protein bands were determined using an Odyssey Imaging System (LI-COR, V3.0) and quantified via ImageJ (V1.8.0).

### 4.10. Enzyme-Linked Immunosorbent Assay

Cardiac troponin I (cTnI, CSB-E08421; CUSABIO, Wuhan, China), IL-1β (RK00006; Abclonal, Wuhan, China), and cytochrome C (Cyt C, CSB-E08532; CUSABIO) levels in cardiac homogenates or cytoplasmic fractions were measured using commercial ELISA kits. Cytosolic fractions were isolated using tissue/cellular mitochondrial isolation kits (C3606/C3601; Beyotime).

### 4.11. Mitochondrial Function Assays

Myocardial mitochondrial Complex I activity [61], malondialdehyde (MDA) and total superoxide dismutase (T-SOD) [62] were quantified using standardized assay kits (BC0515, Solarbio, Beijing; E-BC-K025 and E-BC-K020, Elabscience, Wuhan, China). Intracellular ROS (DCFH-DA, S0033; Beyotime), mitochondrial ROS (MitoSOX, M36007; Invitrogen), mPTP opening (calcein-AM, C2009S; Beyotime) were assessed in H9c2 cells via fluorescence microscopy. ATP content was quantified using the Enhanced ATP Assay Kit (S0027; Beyotime) via luminescence measurement.

### 4.12. Isolation of Mitochondria-Depleted Cytosol Fractions

Myocardial or cellular cytosol component without mitochondria was isolated using Tissue or Cell Mitochondrial Isolation Kit (C3606 or C3601; Beyotime) following the manufacturer’s instructions [62]. Briefly, the lysed cardiac tissue or H9c2 cells were performed to differential gradient centrifugation at 1000× *g* and 11,000× *g* to isolate distinct subcellular fractions, including the nuclear, mitochondrial, and mitochondria-depleted cytosol components. To minimize mitochondrial contamination, the supernatant obtained after the 11,000× *g* centrifugation was subjected to an additional centrifugation at 12,000× *g* for 10 min.

### 4.13. Extraction of Genomic DNA and Real-Time Quantitative PCR (qPCR) Analysis

The mitochondria-depleted cytosolic fractions were immediately transferred to sterile Eppendorf tubes. Total DNA was extracted and purified using a Genomic DNA Small Extraction Kit (D0063; Beyotime). DNA concentrations were determined with Qubit 1 × dsDNA HS Assay Kits (Q33230; Invitrogen, Carlsbad, CA, USA). The relative expressions of cytosolic mtDNA (*mt-Cytb* primer, Invitrogen) and mitochondrial complex I-related genes (*Ndufv2*, *Ndufs1* and *Ndufs4*, Invitrogen) were quantified with the qPCR system (ABI 7500) [63,64,65]. All primer sequences were provided in Table S3. The relative expression levels were normalized to *18S* or *Gapdh*.

### 4.14. Immunofluorescence Co-Localization of Mitochondria and dsDNA

In accordance with established methodologies [34,35], H9c2 cells were dual-stained with 0.2 μM Mito-Tracker Red CMXRos (C1035; Beyotime) and PicoGreen dsDNA (HB220615; Yeason, Shanghai, China). Thereafter, fluorescence co-localization was visualized by fluorescence microscopy (ECLIPSE Ti2-U, Nikon, Tokyo, Japan).

### 4.15. Statistical Analysis

Sample size was estimated based on effect sizes from previous literature to achieve statistical significance (α = 0.05) with at least 80% power (β = 0.20). Data (mean ± SEM) were analyzed using Prism 9.5.0. Group differences were assessed by the Two-tailed unpaired *t* test, one-way with Dunnett’s multiple comparisons test and two-way ANOVA with Sidak’s post hoc analysis. In this study, */# *p* < 0.05; **/## *p* < 0.01; ***/### *p* < 0.001; ns, not significant. The number of independent repeats (n) is indicated in the figure legends.

## 5. Conclusions

In summary, our work provides compelling evidence that the ROS-BAX-mtDNA axis is a critical driver of METH/STS-induced cardiomyocyte pyroptosis. METH facilitated mtDNA leakage by inducing mitochondrial respiratory chain complex I-related oxidative stress and BAX pore formation, thereby triggering caspase-1/GSDMD-dependent cardiomyocyte pyroptosis. Importantly, we demonstrated that the mitochondria-targeted antioxidant mitoQ effectively attenuated this pathological cascade by suppressing the ROS/BAX/mtDNA axis.

## Data Availability

The original contributions presented in this study are included in the article/Supplementary Material. Further inquiries can be directed to the corresponding authors.

## Supplementary Materials

The following supporting information can be downloaded at: https://www.mdpi.com/article/10.3390/ijms27115000/s1.

## Abbreviations

METH	Methamphetamine
STS	Subthreshold thermal stress
NC	Normothermic condition
cTnI	Cardiac troponin I
EF	Ejection Fraction
FS	Fractional Shortening
mtDNA	Mitochondrial DNA
Cyt C	Cytochrome C
ROS	Reactive oxygen species
MitoQ	Mitoquinone mesylate
