# Supplementary material for: Subthreshold Thermal Stress Aggravates Methamphetamine-Induced Cardiomyocyte Pyroptosis via the Mitochondrial ROS/BAX/mtDNA/NLRP3 Pathway
Source: Int J Mol Sci. 2026 May 31;27(11):5000. doi: 10.3390/ijms27115000 (PMC13256843; doi:10.3390/ijms27115000)
Supplement: Supplementary file 1 [file ijms-27-05000-s001.zip › Supplementary Material.pdf]

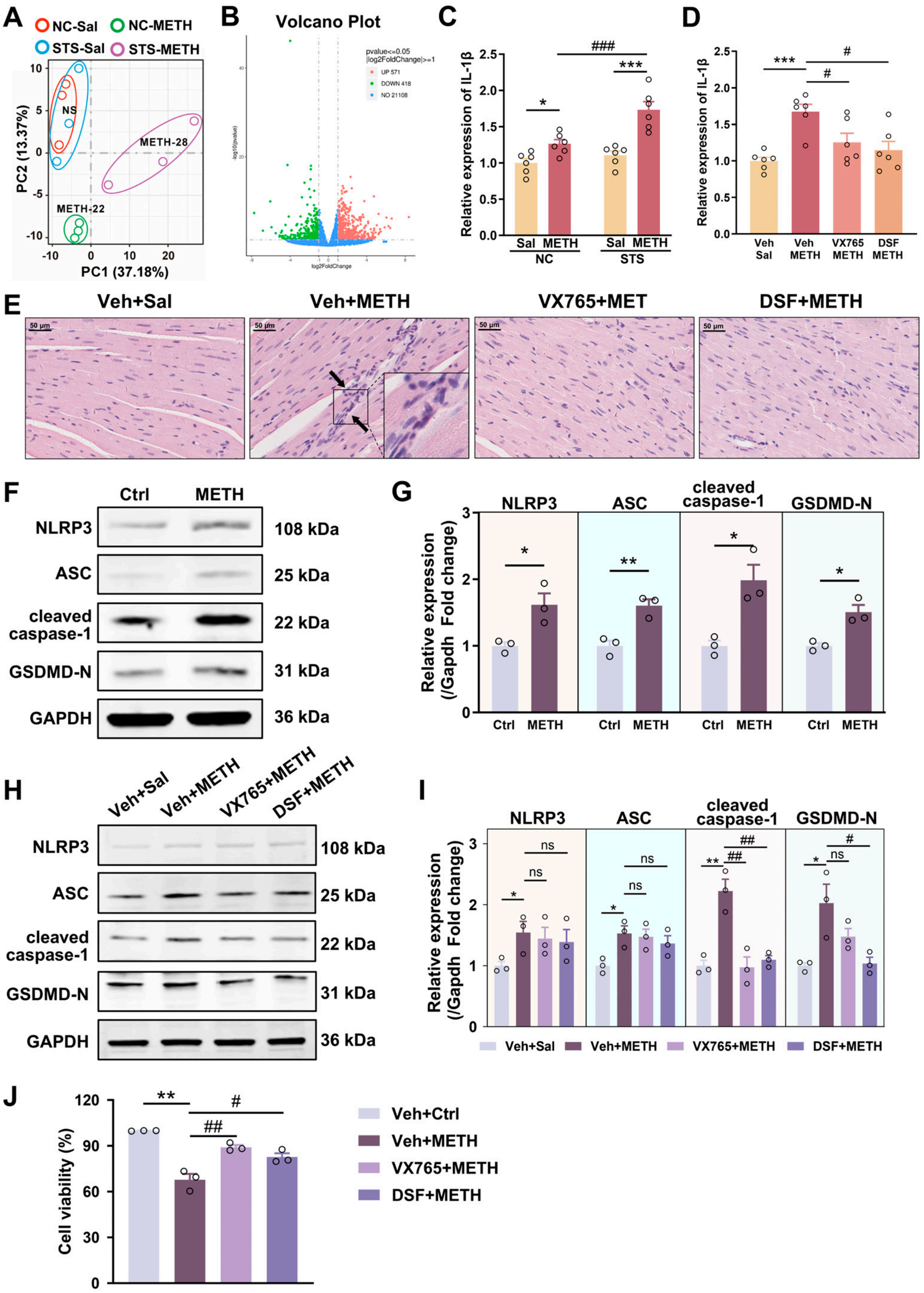

Figure. S1. METH/STS triggers cardiomyocyte pyroptosis in vivo and in vitro. (A) Principal component analysis (PCA) between the NC-Sal, STS-Sal, NC-METH and STS-METH groups. (B) Volcano plots between STS-Sal and STS-METH groups. Myocardial IL-1β level (C; n = 6) under different conditions. Myocardial IL-1β levels (D; n = 6) and representative HE staining (E; scale bar = 50 μm) after STS/METH exposure with or without pyroptosis inhibitor pretreatment (VX765 or DSF). Representative im-ages of pyroptosis effector proteins expression (F-G; n = 3) in H9c2 cells exposed to METH. Representative images of pyroptosis effector proteins expression (H-I; n = 3) and cell viability (J; n = 3) and in H9c2 cells exposed to METH with VX765 or DSF pre-treatment.

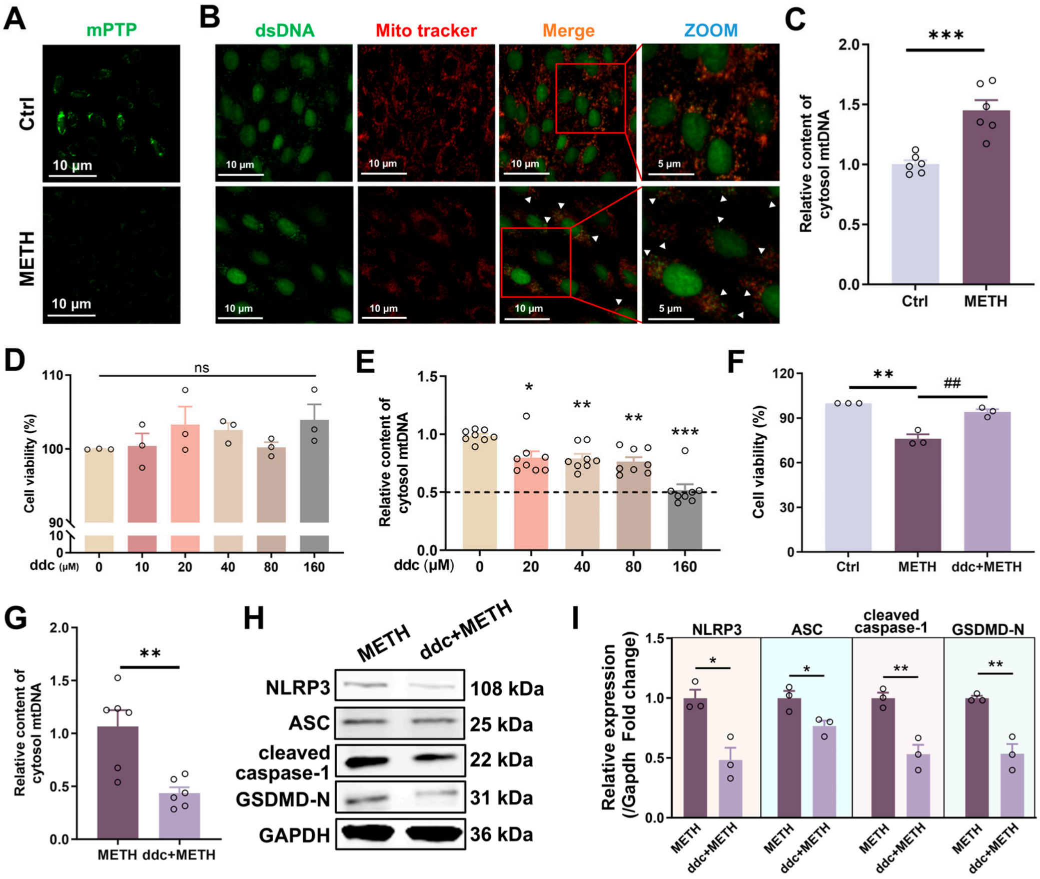

Figure. S2. METH/STS triggers cardiomyocyte pyroptosis via mtDNA escape. The alterations of mPTP channel opening (A; scale bar = 10 μm), dsDNA/mitotracker red double fluorescent staining (B; scale bar = 10/5 μm) and cytosol mtDNA content (C; n = 6) in METH/STS-challenge H9c2 cells. Cell viability (D; n = 3) and cytosol mtDNA content (E; n = 8) exposed to incremental doses of ddc. Cells viability (F; n = 3), cytosol mtDNA content (G; n = 6) and pyroptosis effector proteins (H-I; n = 3) in H9c2 cells following METH/STS exposure with/without ddc pretreatment.

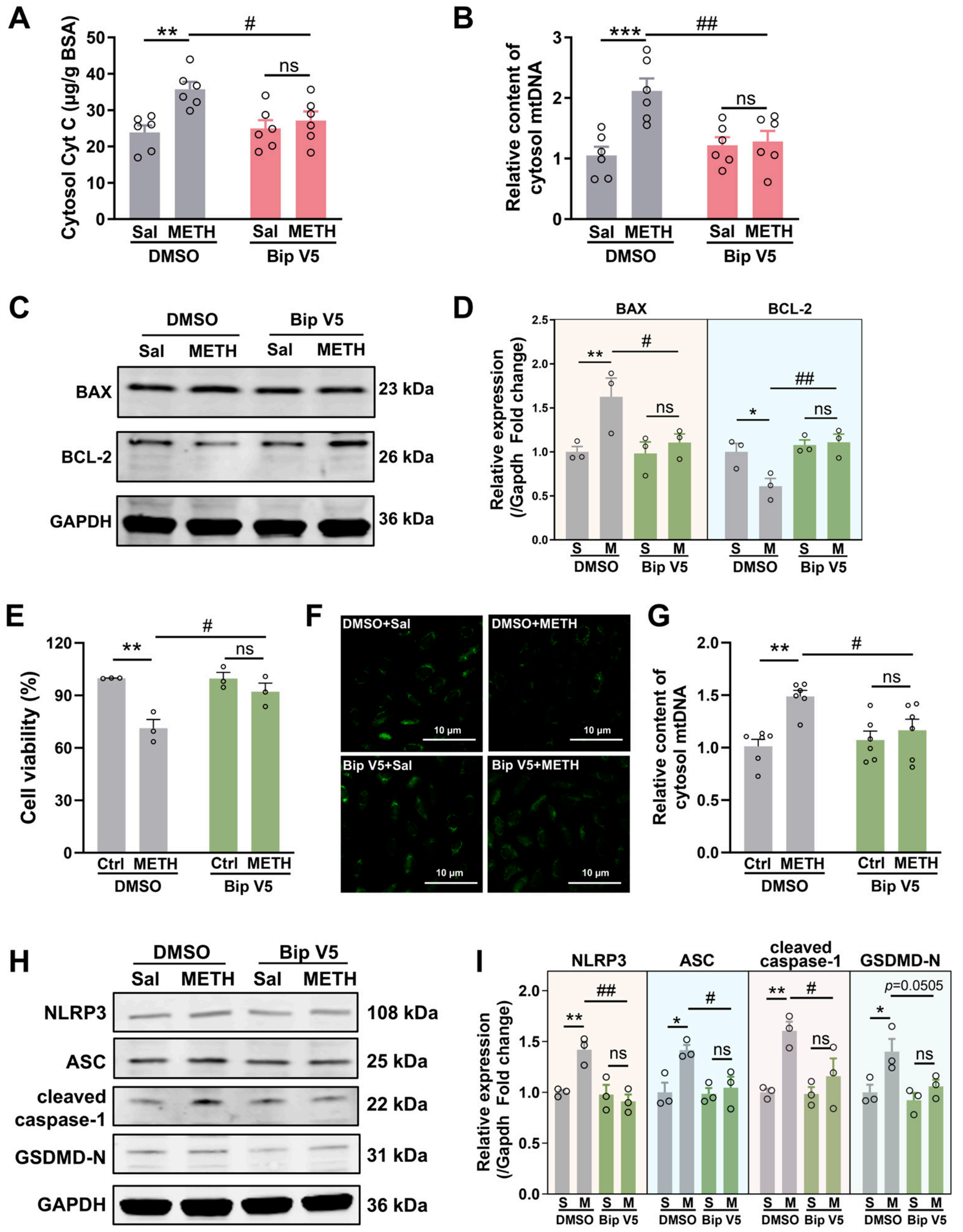

Figure. S3. METH/STS activates cardiomyocyte pyroptosis via BAX-mediated mtDNA escape. Quantitative analysis of cytosol Cyt C and mtDNA content (A-B; n = 6) after METH/STS exposure with or without Bip V5 pretreatment in vivo. Western blot analysis of BCL-2-related proteins expression (C-D; n = 3), cell viability (E; n = 3), the alterations of mPTP channel opening (F; scale bar = 10 μm), cytosol mtDNA content (G; n = 6) and pyroptosis effector proteins expression (H-I; n = 3) in the METH/STS-exposed H9c2 cells with/without Bip V5 pretreatment.

Table S1. Gene ontology of RNAseq data (*p* value, Counts)

Description	NC-Sal *vs* NC-METH	STS-Sal *vs* STS-METH	NC-METH *vs* STS-METH
- Regulation of inflammatory response	0.7717362 (9)	1.339E-08 (47)	1.187E-06 (37)
- Production of molecular mediator involved in inflammatory response	0.7825885 (2)	0.0603208 (9)	0.0822331 (8)
- Response to interleukin-1	0.4894203 (6)	2.145E-06 (23)	0.0036320 (15)
- Interleukin-1 beta production	0.2859865 (6)	0.0088231 (12)	0.0110849 (8)

Table S2. Gene ontology of RNAseq data

Description	Count	*p* value
- Positive regulation of release of cytochrome c from mitochondria	5	0.00046
- Regulation of mitochondrial membrane potential	6	0.01268
- Mitochondrial outer membrane permeabilization	3	0.03410
- Apoptotic mitochondrial changes	8	0.00596
- Mitochondrial outer membrane permeabilization involved in programmed cell death	3	0.05107

Table S3. The sequences of primers (SYBR) for qPCR (Invitrogen, USA)

Species	Gene	Sequences(5'-3')
Mouse	*mt-Cytb*	F: TGTCGGACGAGGCTTATATTATGG
		R: TGTGGCTATGACTGCGAACAG
Mouse	*18S*	F: CTTAGAGGGACAAGTGGCG
		R: ACGCTGAGCCAGTCAGTGTA
Rat	*mt-Cytb*	F: ATTCCGCCCAATCACCCAAATCC
		R: GTTCTACTGGTTGGCCTCCGATTC
Rat	*18S*	F: TAGAGGACAAGTGGCGTTC
		R: CATACTCAGCACCAGCAT
Mouse	*Ndufv2*	F: GCAAGGAATTTGCATAAGACAGC
		R: TAGCCATCCATTCTGCCTTTG
Mouse	*Ndufs1*	F: AGGATATGTTCGCACAACTGG
		R: TCATGGTAACAGAATCGAGGGA
Mouse	*Ndufs4*	F: CGGCGGTCTCAATGTCAGTGTC
		R: GAAGGTCAGAACCATGTTGGAGAGG
Mouse	*Gapdh*	F: GGAGAAACCTGCCAAGTATGA
		R: TTGAAGTCACAGGAGACAACC
