# Supplementary material for: Subthreshold Thermal Stress Aggravates Methamphetamine-Induced Cardiomyocyte Pyroptosis via the Mitochondrial ROS/BAX/mtDNA/NLRP3 Pathway
Source: Int J Mol Sci. 2026 May 31;27(11):5000. doi: 10.3390/ijms27115000 (PMC13256843; doi:10.3390/ijms27115000)

Figure 2D

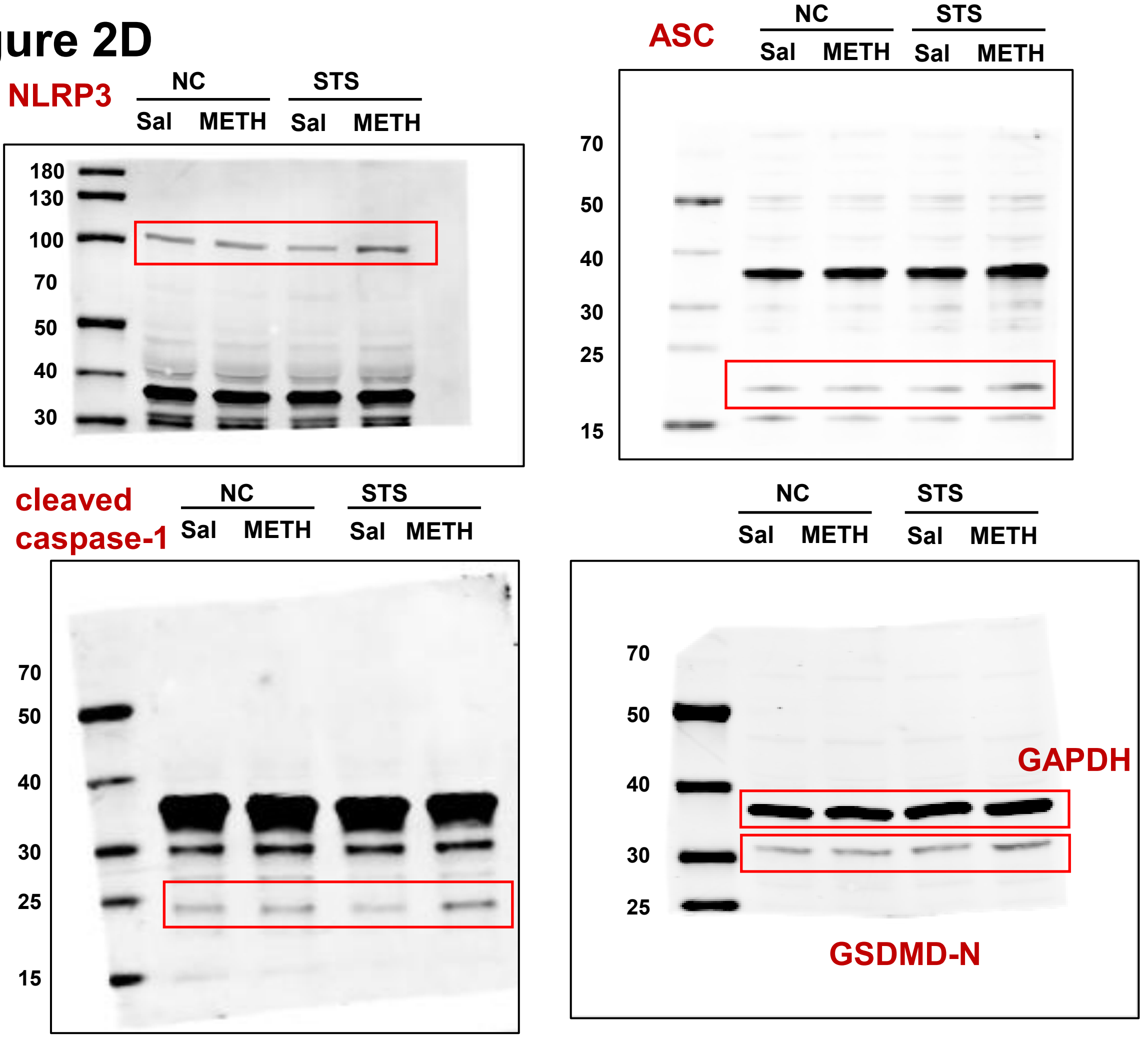

Figure 2F

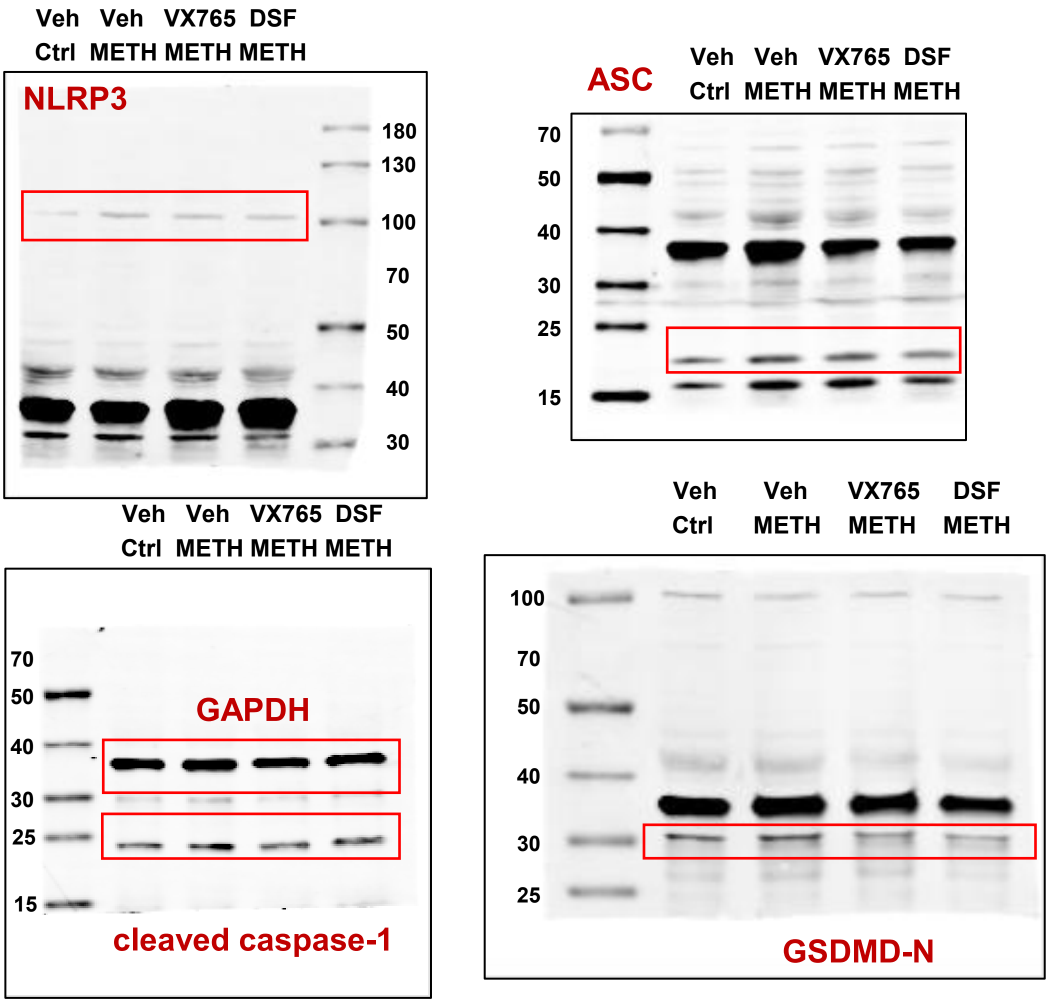

Figure 3I

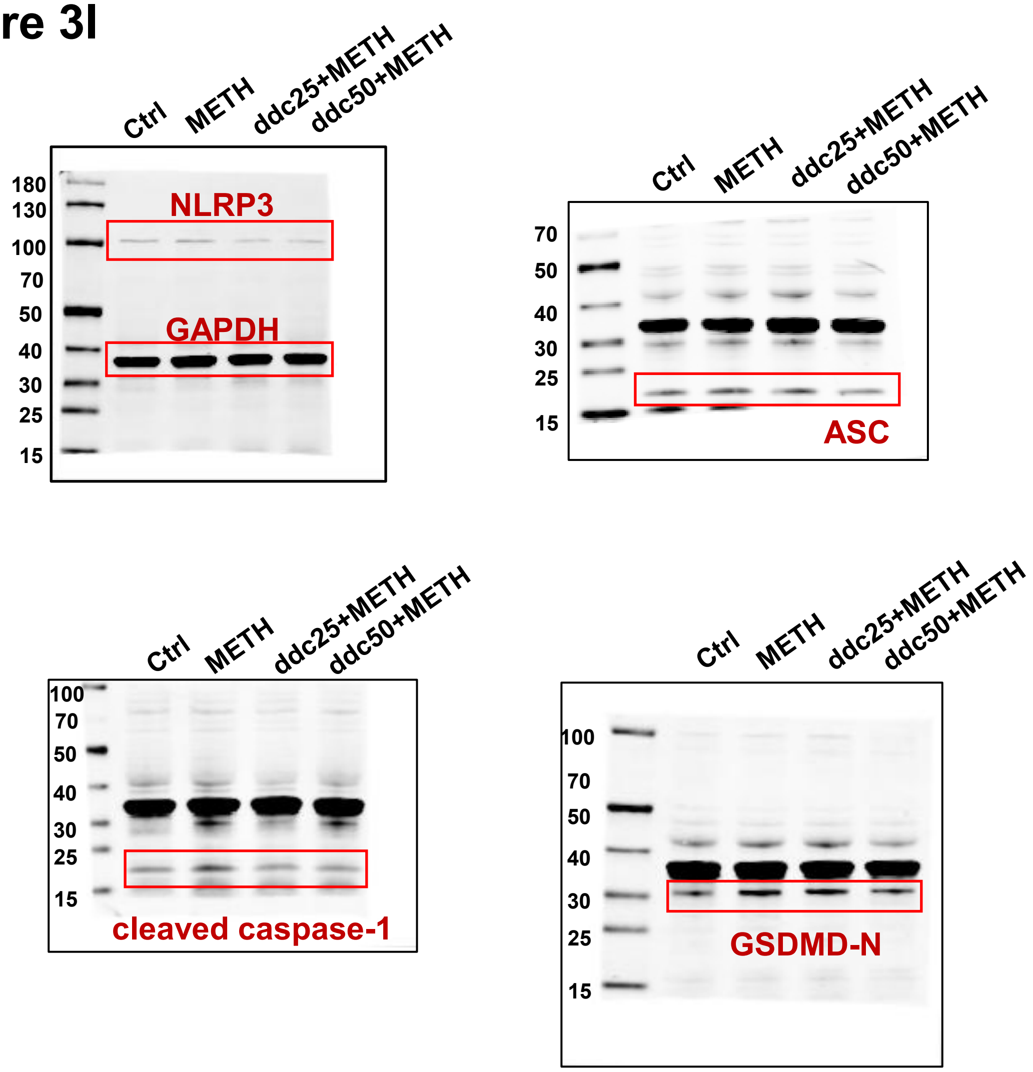
Ctrl
METH
ddc25+METH
ddc50+METH
180
130
100
70
50
40
30
25
15
NLRP3
GAPDH
Ctrl
METH
ddc25+METH
ddc50+METH
70
50
40
30
25
15
ASC
Ctrl
METH
ddc25+METH
ddc50+METH
100
70
50
40
30
25
15
cleaved caspase-1
Ctrl
METH
ddc25+METH
ddc50+METH
100
70
50
40
30
25
15
GSDMD-N

Figure 4B

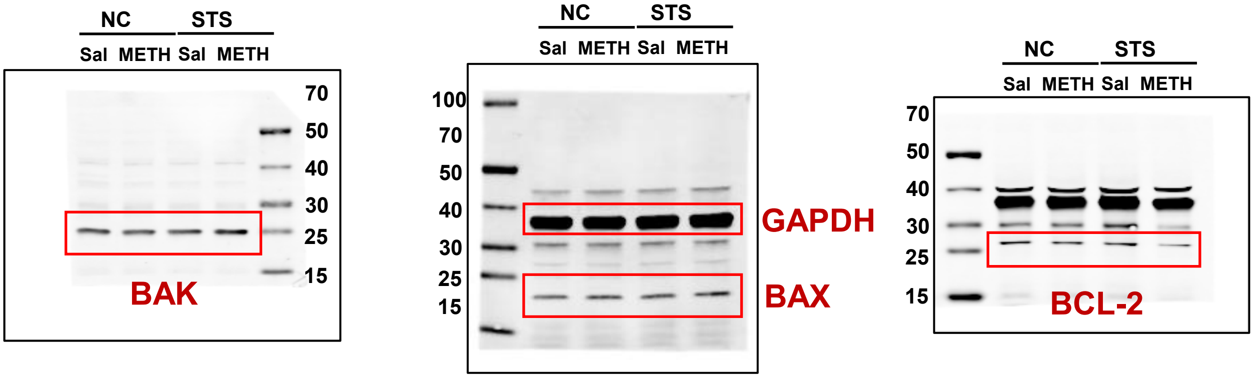

Figure 4D

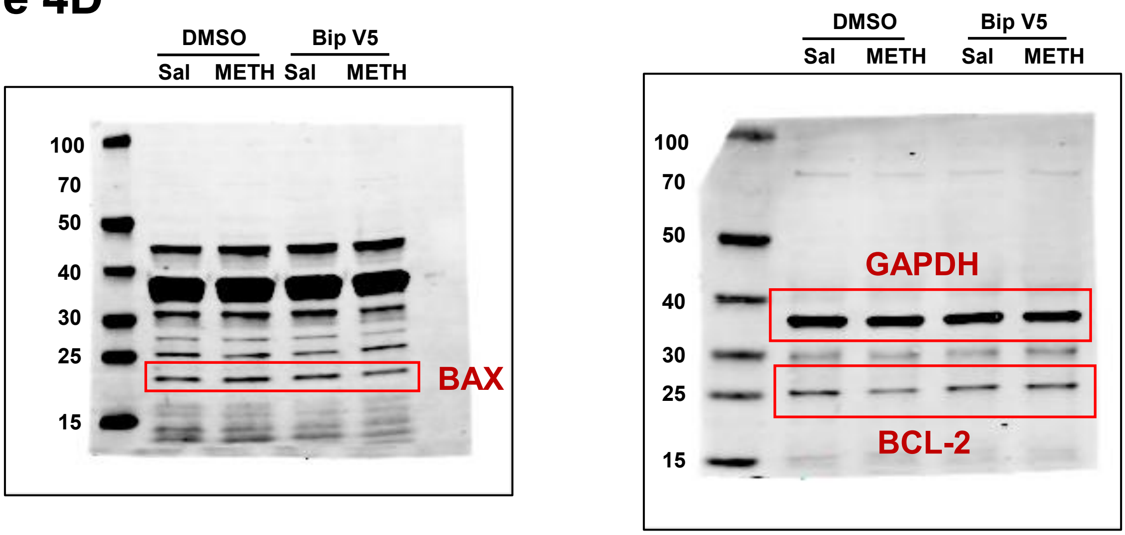

Figure 4J

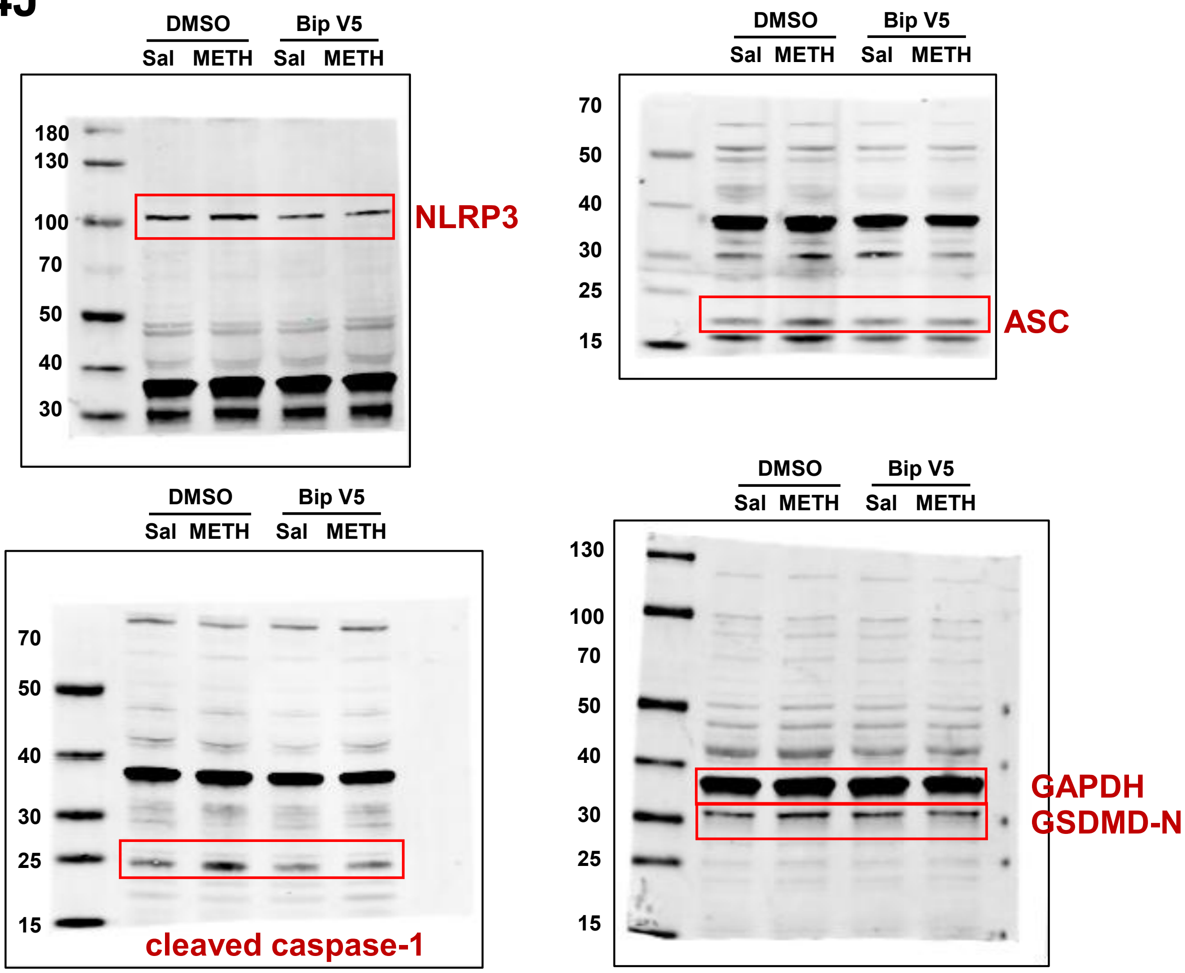

Figure 6D

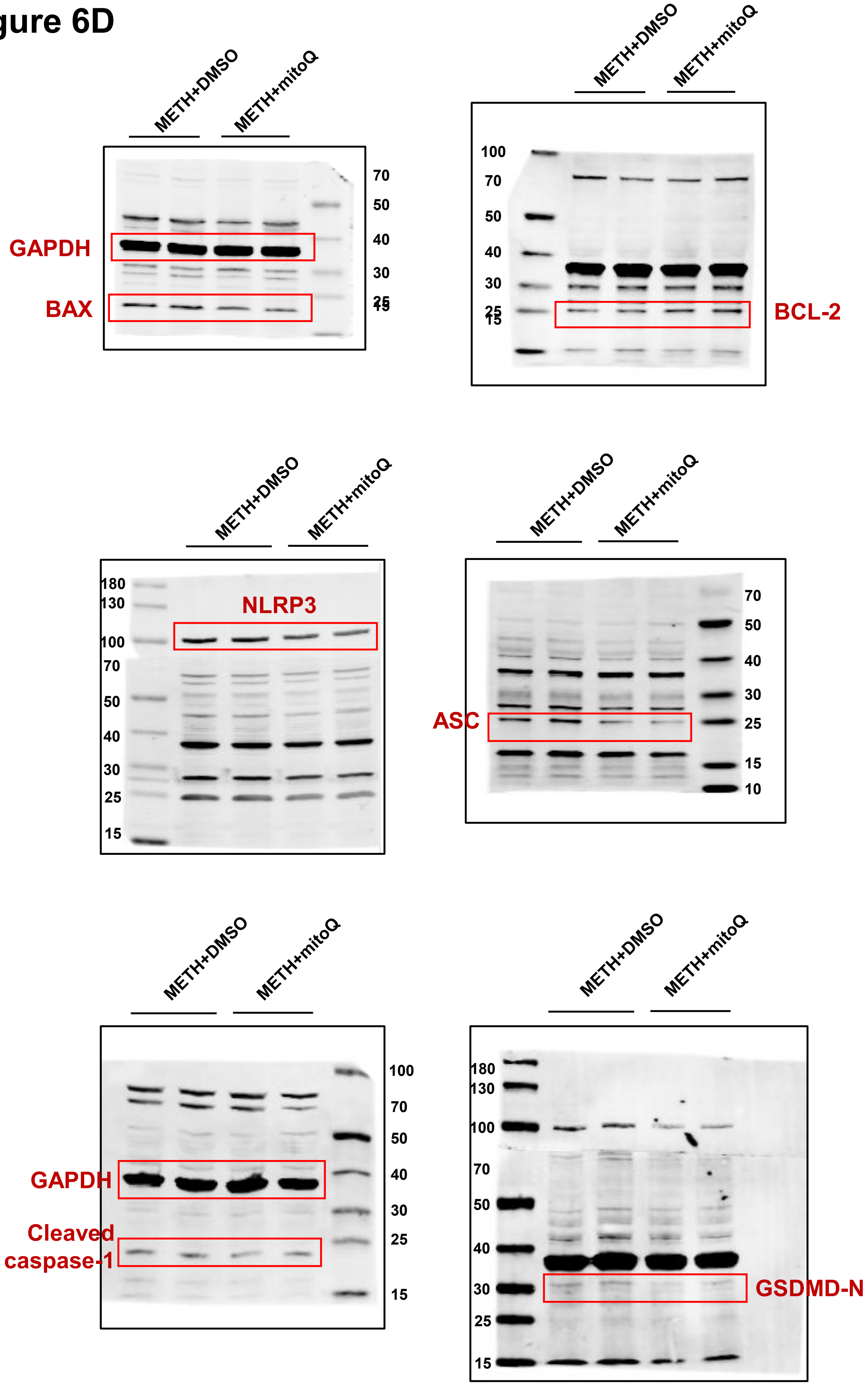

METH+DMSO
METH+mitoQ
GAPDH
BAX
70
50
40
30
25
15
BCL-2
100
70
50
40
30
25
15
NLRP3
180
130
100
70
50
40
30
25
15
ASC
70
50
40
30
25
15
10
GAPDH
Cleaved caspase-1
100
70
50
40
30
25
15
GSDMD-N
180
130
100
70
50
40
30
25
15

Figure 6K

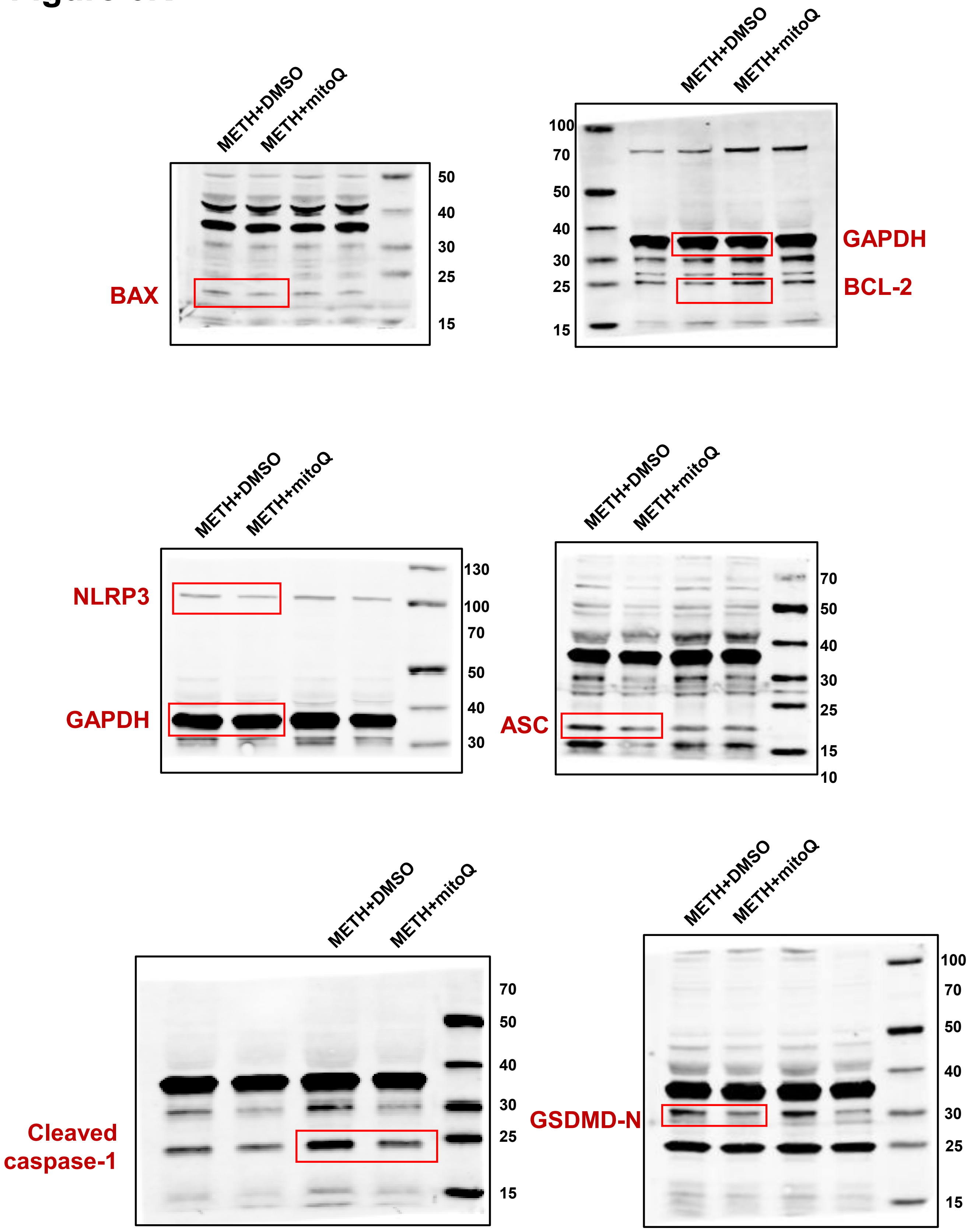

METH+DMSO
METH+mitoQ
BAX
50
40
30
25
15
METH+DMSO
METH+mitoQ
100
70
50
40
30
25
15
GAPDH
BCL-2
METH+DMSO
METH+mitoQ
NLRP3
GAPDH
130
100
70
50
40
30
METH+DMSO
METH+mitoQ
ASC
70
50
40
30
25
15
10
METH+DMSO
METH+mitoQ
Cleaved caspase-1
70
50
40
30
25
15
METH+DMSO
METH+mitoQ
GSDMD-N
100
70
50
40
30
25
15

Figure S1F

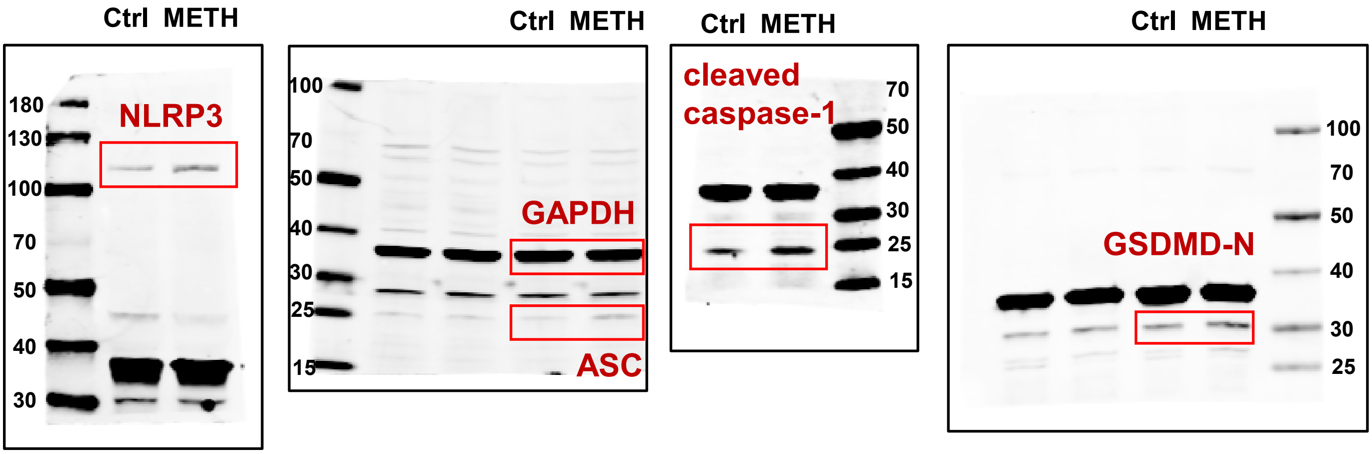

Figure S1H

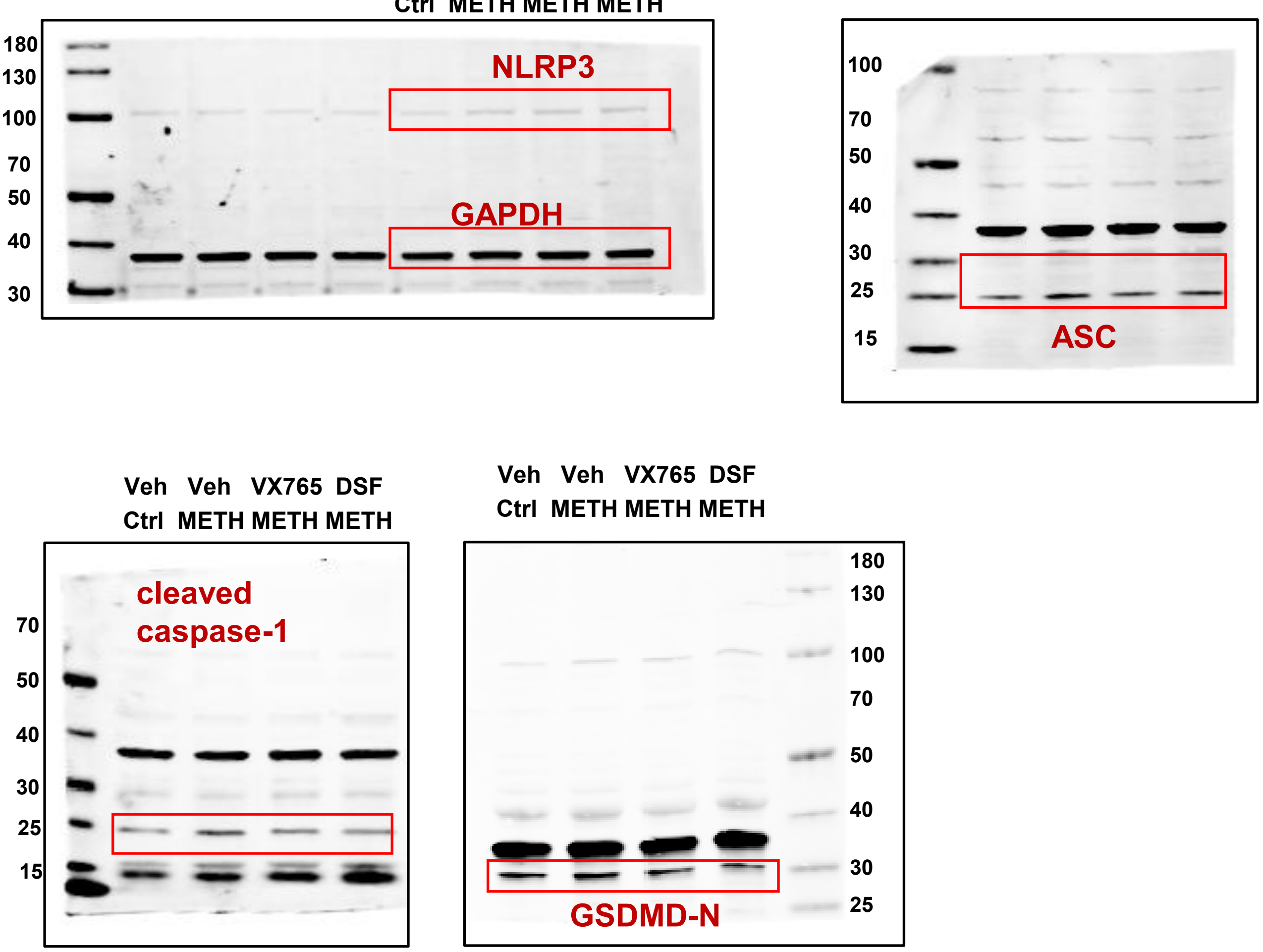

Figure S2H

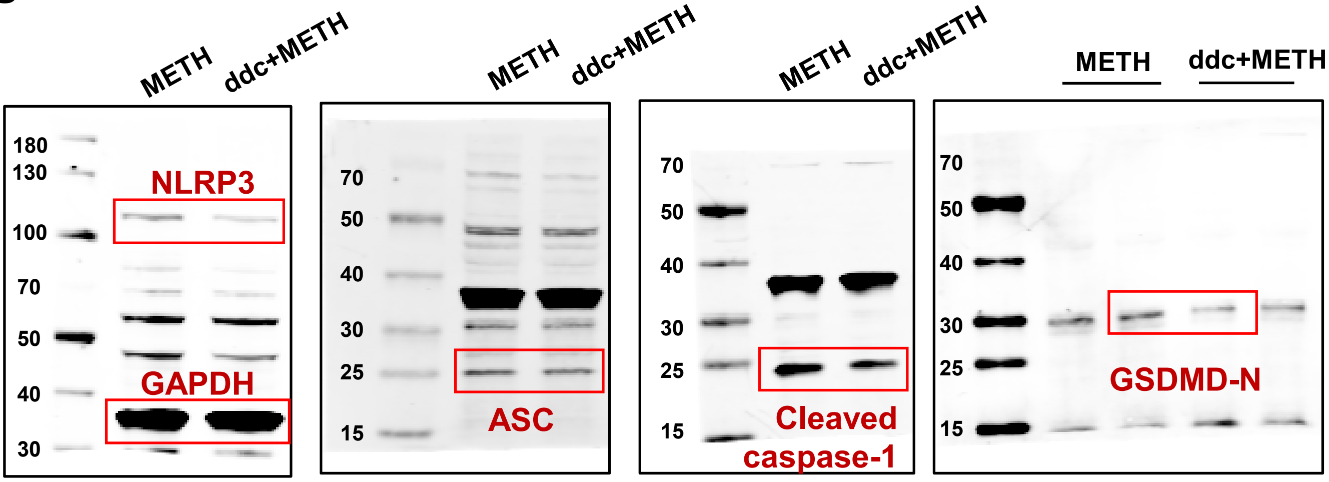

Figure S3C

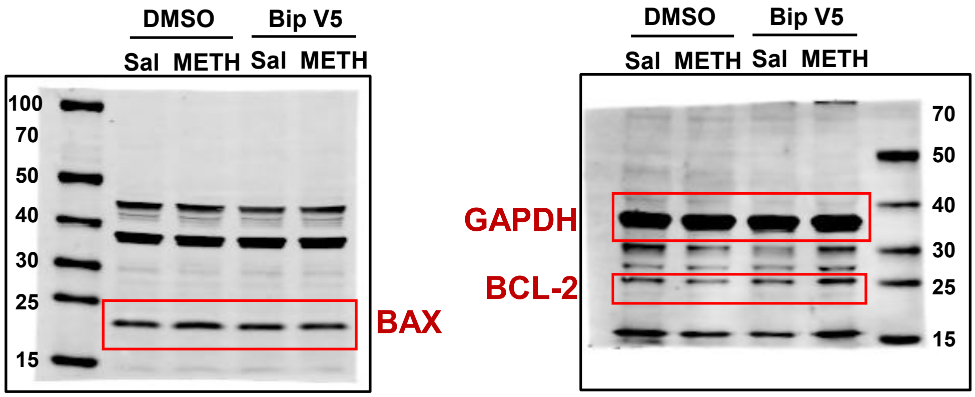

Figure S3H

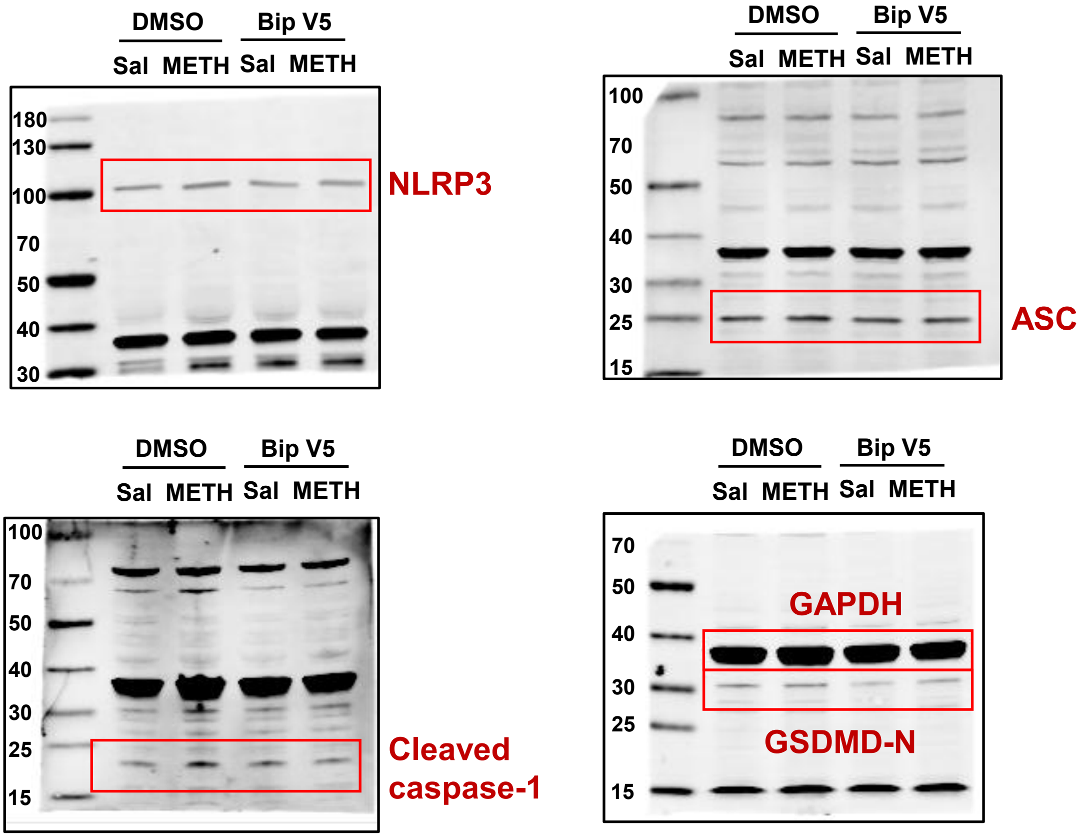

Supplement: Supplementary file 1 [file ijms-27-05000-s001.zip › Western Blot.pdf]
