# Supplementary figures and images for: Subthreshold Thermal Stress Aggravates Methamphetamine-Induced Cardiomyocyte Pyroptosis via the Mitochondrial ROS/BAX/mtDNA/NLRP3 Pathway
Source: Int J Mol Sci. 2026 May 31;27(11):5000. doi: 10.3390/ijms27115000 (PMC13256843; doi:10.3390/ijms27115000)

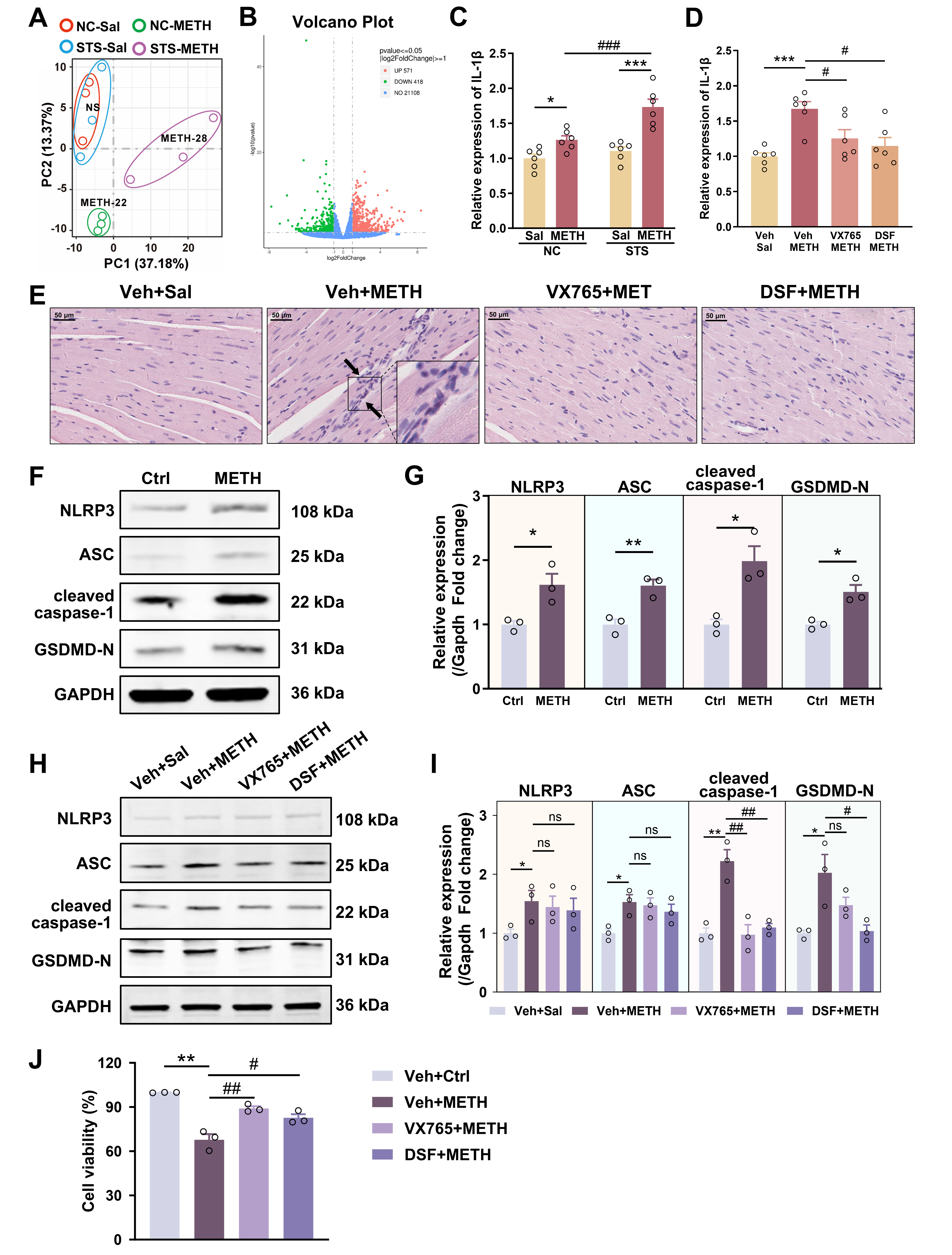

Supplement: Supplementary file 1 [file ijms-27-05000-s001.zip › Figure S1.TIF]

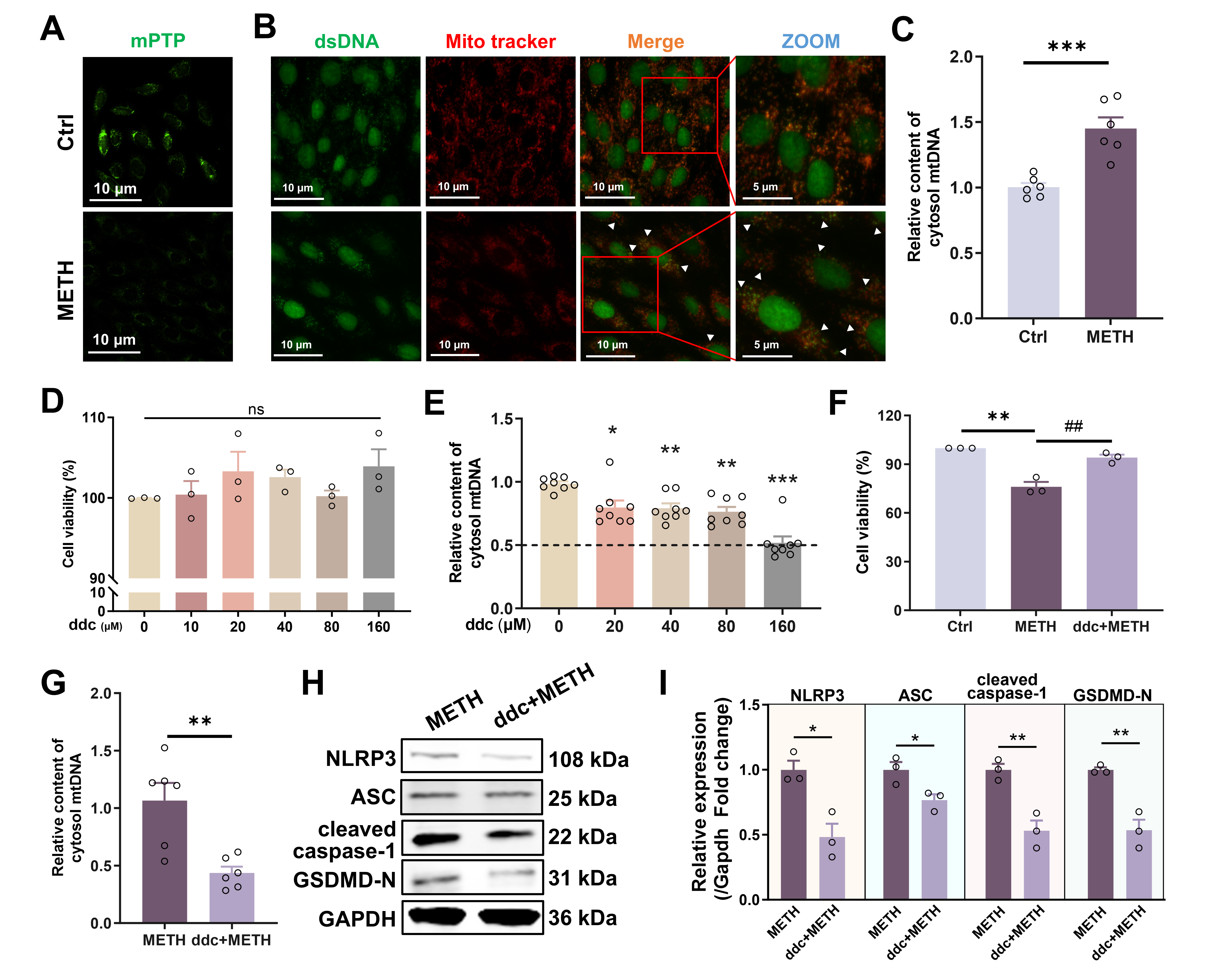

Supplement: Supplementary file 1 [file ijms-27-05000-s001.zip › Figure S2.TIF]

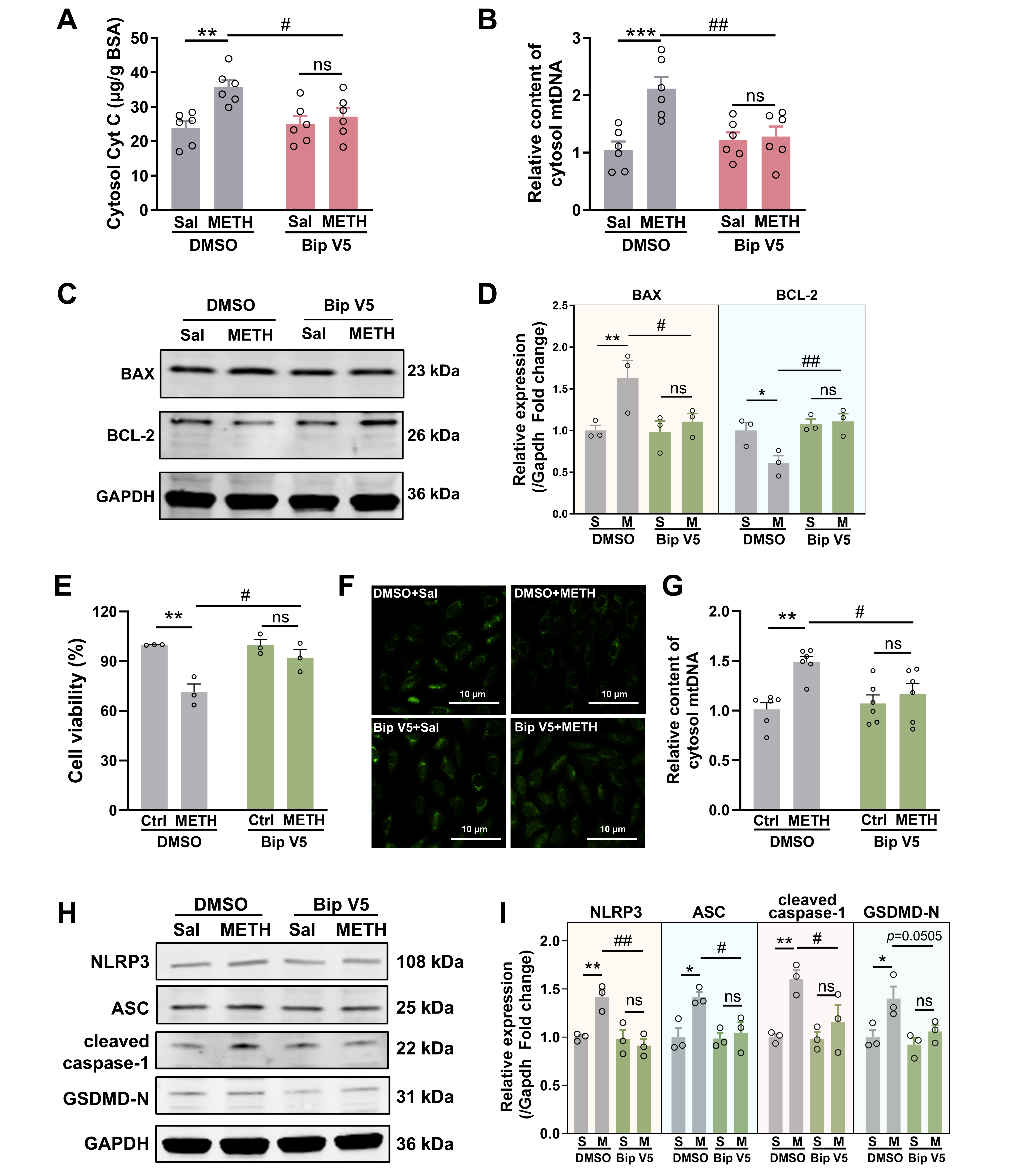

Supplement: Supplementary file 1 [file ijms-27-05000-s001.zip › Figure S3.TIF]
